# Interplay between eutrophication and climate warming on bacterial communities in coastal sediments differs depending on water depth and oxygen history

**DOI:** 10.1038/s41598-021-02725-x

**Published:** 2021-12-03

**Authors:** Laura Seidel, Elias Broman, Stephanie Turner, Magnus Ståhle, Mark Dopson

**Affiliations:** 1 Centre for Ecology and Evolution in Microbial Model Systems (EEMiS), Linnaeus University, Kalmar, Sweden; 2 Department of Ecology, Environment and Plant Sciences, Stockholm University, Stockholm, Sweden; 3 Baltic Sea Centre, Stockholm University, Stockholm, Sweden

**Keywords:** Climate sciences, Environmental sciences

## Abstract

Coastal aquatic systems suffer from nutrient enrichment, which results in accelerated eutrophication effects due to increased microbial metabolic rates. Climate change related prolonged warming will likely accelerate existing eutrophication effects, including low oxygen concentrations. However, how the interplay between these environmental changes will alter coastal ecosystems is poorly understood. In this study, we compared 16S rRNA gene amplicon based bacterial communities in coastal sediments of a Baltic Sea basin in November 2013 and 2017 at three sites along a water depth gradient with varying bottom water oxygen histories. The shallow site showed changes of only 1.1% in relative abundance of bacterial populations in 2017 compared to 2013, while the deep oxygen-deficient site showed up to 11% changes in relative abundance including an increase of sulfate-reducing bacteria along with a 36% increase in organic matter content. The data suggested that bacterial communities in shallow sediments were more resilient to seasonal oxygen decline, while bacterial communities in sediments subjected to long-term hypoxia seemed to be sensitive to oxygen changes and were likely to be under hypoxic/anoxic conditions in the future. Our data demonstrate that future climate changes will likely fuel eutrophication related spread of low oxygen zones.

## Introduction

The world’s coastal oceans are important ecosystems that are increasingly degraded through human influence^1^. Two of the greatest threats to worldwide coastal marine health are accelerated eutrophication caused by anthropogenic nutrient inputs^2^ and anthropogenic induced climate change, including for example increased temperature of the water column, stratification, oxygen decline, and acidification^3–5^. However, to what extent many years of eutrophication and ongoing climate change will alter coastal oceans globally is poorly understood.

The Baltic Sea in northern Europe is heavily stressed by human activities including increased CO_2_ levels, elevated temperature, nutrient input, change in the distribution and abundance of marine species, as well as deoxygenation^4,6^. For example, warming of the Baltic Sea has resulted in shorter sea-ice seasons and reduced ice-thickness within the last century^7^. Modelling of Baltic Sea surface temperature predicts a likely rise of 2–4 °C by the end of this century^8^, resulting in an approximate reduction of sea-ice by 50–80%^7^. Warming of northern hemisphere water bodies, like the Baltic Sea, has accelerated two to four times greater than the global mean rate^9^ and how the seas respond is strongly influenced by interactions with other human-driven changes on the marine system, including overfishing and industrial plus household discharges^10,11^. These effects might result in a more fragile marine ecosystem, although to what extent this will happen remains uncertain.

Anthropogenic influenced increased nutrient concentrations have led to accelerated eutrophication in the Baltic Sea^12^ while climate warming within the last two decades has worsened the effects^13,14^. Prolonged higher temperatures will likely further accelerate these eutrophication effects such as hypoxic (O_2_ < 2 mg/L) or anoxic (O_2_ = 0 mg/L) conditions in coastal bottom waters, which are derived from organic matter decomposition^15^. These so called ‘dead zones’ have increased ten-fold compared to 100 years ago in the Baltic Sea^13^. In addition, hypoxia/anoxia at the water–sediment interface leads to release of hydrogen sulfide from the sediment that is toxic for benthic life^15^. The Helsinki Commission (HELCOM) proposed routines to reduce nutrient intake that has led to a decrease in nitrogen concentration since 1990 but less improvement in phosphorus reflecting its higher degree of storage in sediments^16^. Various mitigation attempts have been performed to reduce nutrient intake and to recover ‘dead zones’, such as biomanipulation, lowering growth of phytoplankton by precipitation of phosphorus, and artificial re-oxygenation of bottom waters^17^. However, how the interplay between nutrient backlog, mitigation of nitrate and phosphate release, and climate change will alter coastal zones is not well understood.

Microorganisms in sediment use a range of electron acceptors for energy conservation, from oxygen with the highest energy efficiency to alternative electron acceptors including nitrate, manganese (IV), iron (III), and sulfate under hypoxic/anoxic conditions^18^ that favors the growth of anaerobic microorganisms^19^. Previous studies within False Bay, Washington show that organic matter degradation is slower under anaerobic conditions^20^, and it has been estimated in laboratory incubation studies of Baltic Sea sediments that organic matter is consumed upon oxygenation to reach levels similar to long-term oxic sediments after approximately three months at 8 °C^14^. Due to sulfate reduction in oxygen depleted sediments, microbial communities are rich in *Gamma*- and *Deltaproteobacteria*^21^ while *Planctomycetes* and *Betaproteobacteria* are more abundant in oxygen rich sediments^22^. In addition, the *Sulfurimonas* and *Arcobacter* genera have been shown to increase in relative abundance upon oxygenation of anoxic sediment^23^, suggesting they are key players in the microbial community upon re-oxygenation. However, this was observed under laboratory incubation experiments^14^ and has not been investigated under field conditions. Microorganisms are key to nutrient cycling and changes in their community structure and ability to respond to eutrophication in combination with climate change might have large effects on the benthic ecosystem.

In this study, we compared coastal sediments in a Baltic Sea basin in November 2013 and November 2017 from three sites with varying oxygen conditions, oxygen histories, and depth^14,23,24^. These include a shallow site that has been under long-term, constant oxic conditions as shown in previous studies^14^; an intermediate site with seasonal hypoxia; and a deep site that was in previous studies all year anoxic/hypoxic^14,23^. Collected data from the area around the sampling sites showed a history of long-term oxygen deficiency^14,23^. The aim was to investigate spatiotemporal differences in sediment nutrient contents and bacterial communities. We hypothesized that the response to possible ongoing climate change warming effects and eutrophication in coastal sediments was mainly dependent on depth and oxygen histories. More specifically, that bacterial communities in shallower coastal sediments are highly affected but better adapted due to sufficient oxygen concentrations to environmental changes, whereas deeper coastal sediments with long-term hypoxic conditions are sensitive to even small environmental perturbations.

## Results

### Sediment and bottom water chemistry

**Figure 1:**
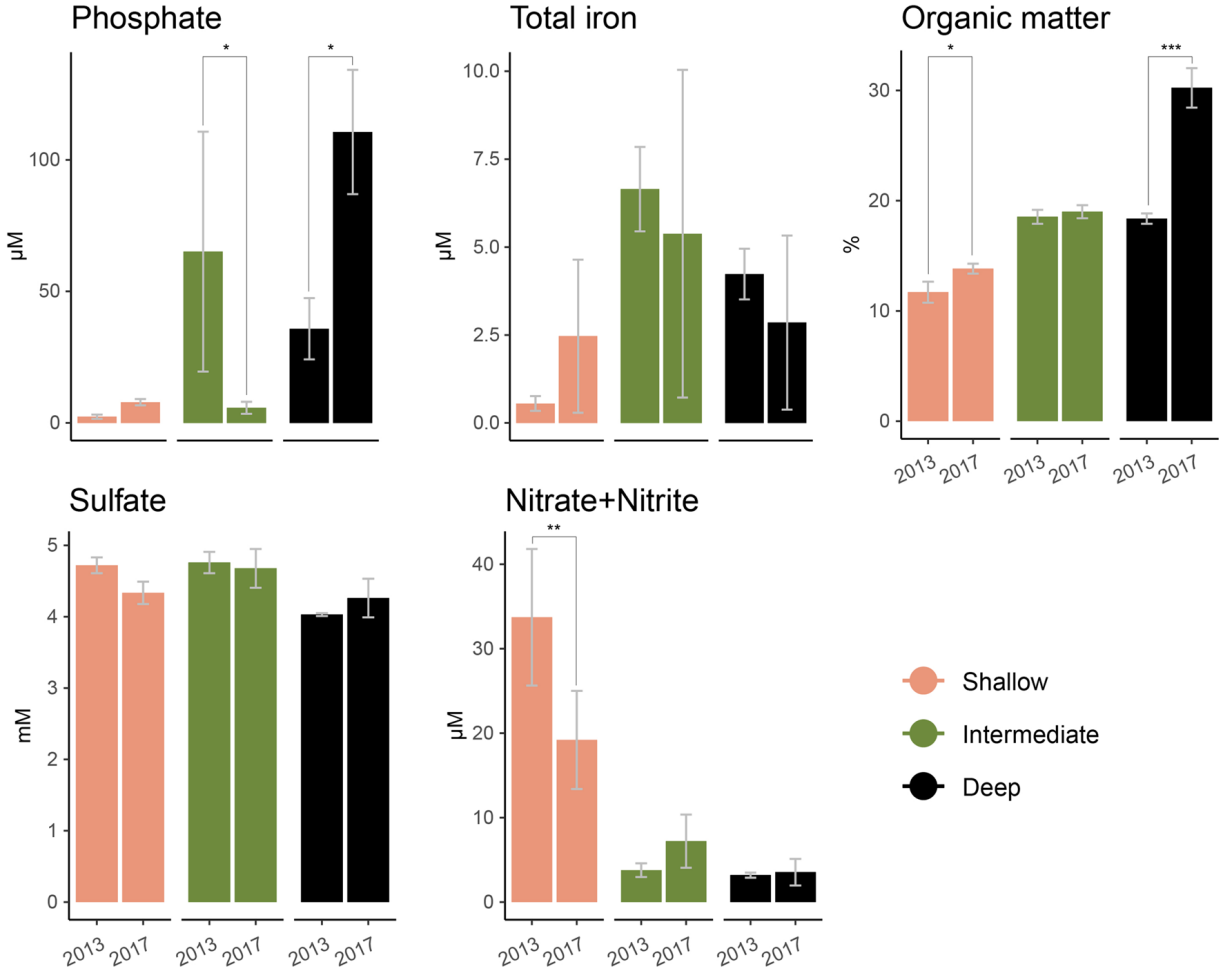
Chemical parameters between 2013 and 2017 in the shallow, intermediate, and deep sites. Chemical parameters were measured on pore water (0–1 cm) for phosphate (µM), total iron (µM), sulfate (mM), and nitrate + nitrite (µM) (in combination) plus on sediment for organic matter (%) in the shallow (salmon), intermediate (green), and deep (black) site in 2013 and 2017. Error bars show the mean and standard deviations (*n* = *3*); significant p-values, ***p < 0.001 **p < 0.01 *p < 0.05.

Within the last two decades data collected by HELCOM and ICES (International Council for the Exploration of the Sea)^25^ showed trends of increasing temperatures on coastal bottom waters (1–30 m below sea surface (mbs)) across the Baltic Sea (Western Gotland-, Eastern Gotland-, and Bornholm-Basins). The temperature increased by several degrees independent of the depth of the coastal bottom waters (Supplemental Fig. S1) along with a trend in oxygen decline (Supplemental Fig. S1). The data within our study showed similar trends (Table 1). The observation station closest to the investigated sampling sites showed a general temperature increase with increasing phosphate and decreasing nitrate concentrations between the years 2001 and 2012 (Supplemental information). Geochemical data from the three sites in 2013 and 2017 in this study are provided in Fig. 1 and Table 1 (statistical support in Supplemental Tables S1; S2) and support the HELCOM data temperature and oxygen concentration trends for the Baltic Sea as a whole.

**Table 1:** Oxygen, temperature, and pH in 2013 and 2017.

Sample	Year	Oxygen (mg/L) *n* = 1	Temperature (°C) *n* = 1	pH *n* = 3	References
Shallow	2013	11.3	6.5	7.85^a^	Broman et al. ^14^
Intermediate	2013	0.8	2.8	7.95^a^	Broman et al. ^14^
Deep	2013	0.85	2.6	8.1 ± 0.19	Broman et al. ^14^
Shallow	2017	5.88	8.5	7.04 ± 0.03	This study
Intermediate	2017	2.55	2.5	6.75 ± 0.04	This study
Deep	2017	0.49	5.5	7.29 ± 0.03	This study

Overview of in situ oxygen and temperature in bottom waters in 2013 and 2017 at each sampling site as well as pH measured for bottom waters for both years and all sites in triplicates; mean (*n* = 1 or 3 as indicated) ± standard deviation.

^a^*n* = 1.

The shallow site bottom water oxygen concentration in November in 2013 was 11.3 mg/L and was lower in 2017 by approximately 50% with 5.88 mg/L (Table 1). At the same time, the bottom water pH dropped from 7.85 to 7.04 (Table 1). The 6.5 m deep shallow site showed a strong geochemical response within the 0–1 cm sediment layer with significantly lower nitrate + nitrite (in combination) concentrations measured in November 2017 (pore water, 2013, *n* = 3, 33.73 ± 8.08 µM compared to 2017, *n* = 3, 19.19 ± 5.81 µM, ANOVA, pairwise comparison, *p* < 0.01) and high variation in total iron as measured in 2017 (pore water, 2013, 0.55 ± 0.21 µM and 2017, 2.47 ± 2.18 µM). Pore water sulfate concentrations were lower in 2017 with 4.33 ± 0.16 mM compared to 4.72 ± 0.11 mM in 2013 while organic matter sediment content was significantly higher (2013, *n* = 3, 11.7 ± 0.95% and 2017, *n* = 3, 13.83 ± 0.45%, *p* < 0.05). The low concentration of pore water phosphate stored in the shallow site had a small increase in 2017 (2013, 2.39 µM ± 0.8 and 2017, 7.86 µM ± 1.20).

The intermediate site showed the smallest changes over time. Contrary to the shallow and deep sites, the intermediate site showed a higher bottom water oxygen concentration in November 2017 with 2.25 mg/L (i.e. above the > 2 mg/L hypoxic cut-off) while being below the cut-off in November 2013 with 0.8 mg/L (Table 1). In addition, the intermediate site showed a lower bottom water pH in 2017 with 6.75 and 7.95 in 2013 (2017, 6.75 ± 0.04, 2013, 7.95; Table 1). Analysis of pore water within 0–1 cm showed nitrate + nitrite concentrations were slightly higher in 2017 (pore water, 2013, 3.78 ± 0.81 µM and 2017, 7.21 ± 3.14 µM) while the pore water total iron concentrations were lower in 2017 with 5.38 ± 4.66 µM compared to 6.65 ± 1.2 µM in 2013. Sulfate concentrations were lower in 2017 (pore water, 4.76 ± 0.15 mM and 4.67 ± 0.27 mM) while organic matter content was slightly increased from 18.54 ± 0.64% in 2013 to 19.00 ± 0.59% in 2017. The strongest response was observed in phosphate concentrations that were significantly lower in 2017 (pore water, 2013, *n* = 3, 65.13 ± 45.62 µM and 2017, *n* = 3, 5.74 ± 2.29 µM, *p* < 0.05).

In contrast to the intermediate site, the deep site sediments had strong responses. Despite that the site was partially oxic in 2017 (Supplemental Fig. S2), it has previously showed long-term hypoxic conditions. In November 2013 the bottom water oxygen concentration was hypoxic with 0.85 mg/L and decreased to 0.49 mg/L (Table 1) in November 2017. The bottom water pH was 8.1 in 2013 and was lower in 2017 with 7.27 (2013, 8.1 ± 0.19, 2017, 7.29 ± 0.03; Table 1). The pore water (0–1 cm) nitrate + nitrite concentration remained low with 3.19 ± 0.3 µM in 2013 compared to 3.54 ± 1.57 µM in 2017 as it was likely continually reduced in this long-term hypoxic site. Pore water total iron was 4.23 ± 0.72 µM in 2013 and showed lower concentrations with 2.85 ± 2.47 µM in 2017. In addition, no significant changes were observed in sulfate concentrations with 4.03 ± 0.02 mM in 2013 and slightly higher concentrations in 2017 (pore water, 4.26 ± 0.27 mM). In contrast, the organic matter content was significantly higher in 2017 (2013, *n* = 3, 18.38 ± 0.47% and 2017, *n* = 3, 30.24 ± 1.79%, *p* < 0.001). Finally, the deep site showed an approximately three-fold higher pore water phosphate concentration in 2017 (2013, *n* = 2, 35.8 ± 11.65 µM and 2017, *n* = 3, 110.59 ± 23.62 µM, *p* < 0.05).

In summary, a general trend of decreasing oxygen concentrations with decreasing depth was observed in 2013 and 2017. Furthermore, while phosphate concentrations peaked within the intermediate site in 2013 they shifted and peaked in 2017 in the deep site (Fig. 1). Similar trends of iron concentrations were observed along the depth gradient in both years, showing highest concentrations at the intermediate site, while organic matter increased with increasing depth (Fig. 1). High nitrate + nitrite concentrations were observed close to the coast and decreased with increasing water depth (Fig. 1).

### Microbial diversity, community composition, and taxonomy

**Figure 2:**
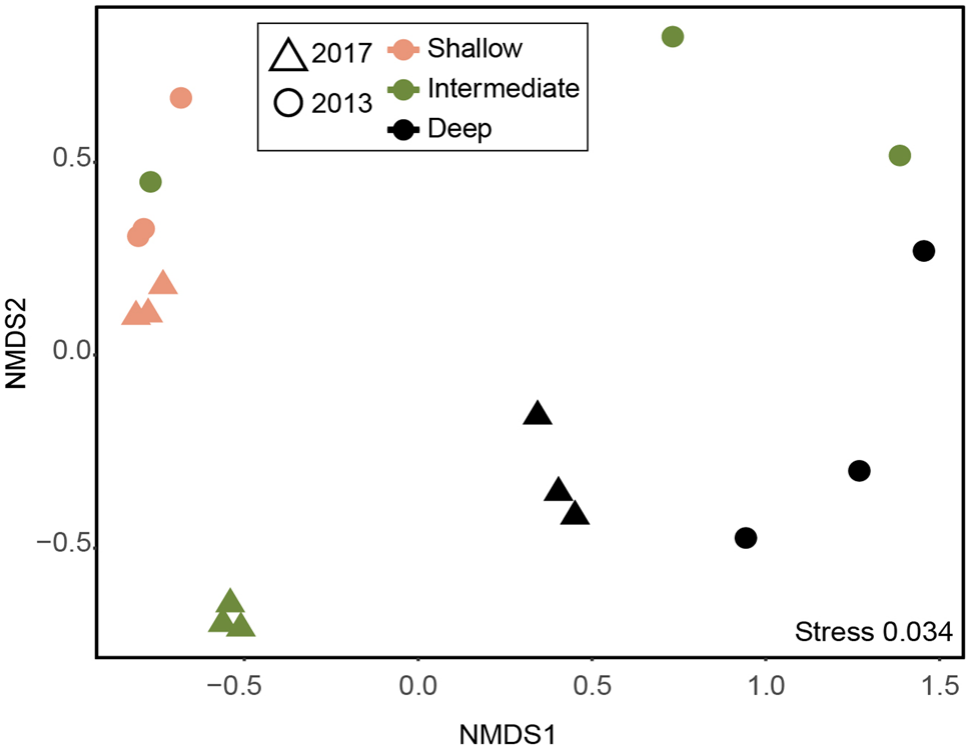
Non-metric multidimensional scaling (nMDS) plot of bacterial sediment communities. NMDS plot showing bacterial communities within each site (*n* = 3) and year (*n* = 2) based on all ASVs. The samples are colored by site (shallow (salmon), intermediate (green), and deep (black)) and years are separated by different symbols (2013 (circle) and 2017 (triangle)).

Rarefying the data to the lowest sample size (Supplemental Table S3 for comparison with rarefied data) confirmed that the main bacterial diversity was covered and therefore, to retain as much data as possible the unrarefied data was used. The unrarefied average sample size was 31,474 reads (*n* = 18; Supplemental Table S4 for filtering results in DADA2). Changes in Shannon’s H diversity occurred in all three sites with a general decrease of diversity with water depth (Supplemental Fig. S3), while the diversity slightly but significantly increased in the intermediate site in 2017 compared to 2013 (ANOVA, posthoc pairwise comparison, *n* = 6, *p* < 0.05; Supplemental Fig. S3 and Supplemental Table S1 for statistical support). The shallow site community composition did not change significantly (PERMANOVA, *n* = 6, *p* > 0.05) from 2013 to 2017 and showed small shifts in the community (Fig. 2, Supplemental Fig. S4, and Supplemental Table S1). The intermediate site community differed from 2013 to 2017 (Fig. 2, Supplemental Fig. S4) although the changes were insignificant (*n* = 6, *p* > 0.05; Supplemental Table S1). The bacterial community composition in the deep site significantly (*n* = 6, *p* < 0.05; Supplemental Table S1) changed comparing 2013 to 2017 (Supplemental Fig. S4), showing shifts and moved towards communities of the two shallower sites (Fig. 2). Finally, the communities in 2017 appeared more similar to each other than was observed in 2013 (Fig. 2 and Supplemental Fig. S4). In line with these results, similarity of percentages analysis (SIMPER) showed high dissimilarities along the depth gradient in 2013 (shallow-intermediate, 71.57%; shallow-deep, 81.25%; deep-intermediate, 74.61%) that decreased in 2017 (shallow-intermediate, 47.72%; shallow-deep, 73.22%; deep-intermediate, 70.76%) (Fig. 2).

The bacterial communities between the different sites along the depth gradient have been shown to be significantly different in a previous study^14^ that was confirmed for the year 2017 (PERMANOVA, p < 0.01, Supplemental Table S1). Based on SIMPER analysis, the major contributors to the differences between the sampling sites in 2013 were the *CG2-30-66-27, UBA6092*, and *Desulfobacula*; while in 2017 the top contributors between sites shifted (Supplementary Tables S4 and Table S5). The top contributors between the shallow and intermediate stayed the same with the *MBNT15* genera *CG2-30-66-27* (6.61%, average contribution of dissimilarity) and *UBA6092* (2.72%), while between the shallow and deep site the main contributor now included the *Cyanobacteria Nodularia* (7.73%). The differences between the deep and intermediate site were *CG2-30-66-27* (10.85%) and *Nodularia* (7.76%) (Supplementary Tables S4 and Table S5).

A general trend could be observed when comparing the bacterial community composition of different sites between the years 2013 and 2017 where the highest dissimilarity were in the deep site (79.29%) with increasing similarity with decreasing depth (intermediate, 60.8%; shallow, 56.66%). Additional constrained ordination analysis (RDA) showed the environmental variables most explaining the changes between the sampling sites and years were oxygen (ANOVA, p < 0.05, Supplemental Table S1 and Supplemental Fig. S5), temperature (p < 0.05), and organic matter (p < 0.01).

The number of detected phyla within each site differed between years, showing a generally higher diversity among the total sum of amplicon sequence variants (ASVs) (Supplemental Fig. S3) but a lower number of abundant ASVs (> 0.5% relative abundance) from 2013 to 2017. The most abundant phyla within each site were the *Proteobacteria*, *Desulfobacterota*, *Bacteroidetes*, and *Cyanobacteria*.

**Figure 3:**
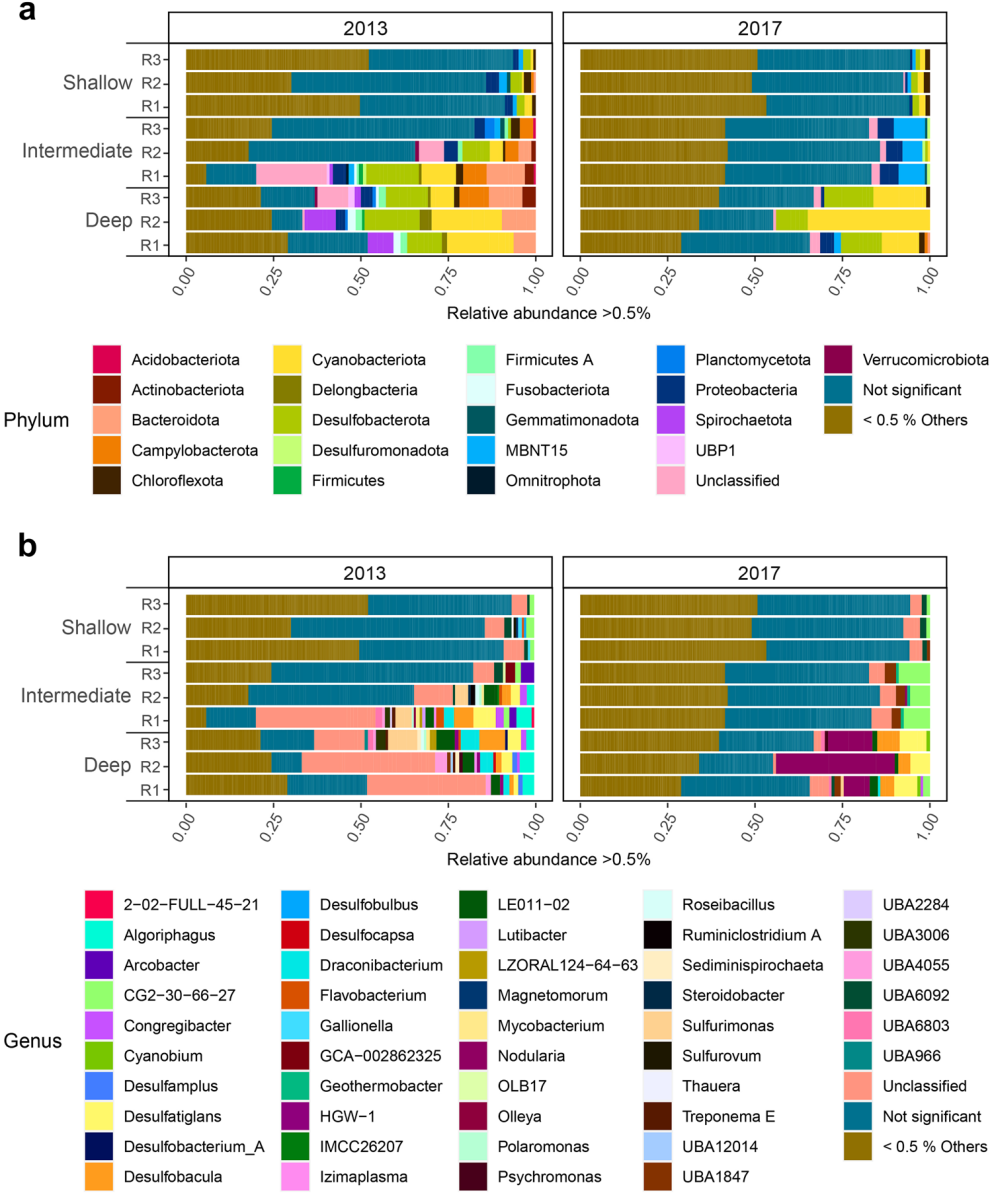
Bar plot of relative abundance on phylum and genus levels. Bar plots of significantly differential abundant taxa over 0.5% relative abundance based on ASVs (the cutoff was based on an ASV having > 0.5% in at least one sample). Phyla < 0.5% and non-significant phyla > 0.5% are summarized. (**a**) Bar plot on phylum level showing years 2013 and 2017 in November at the three different sites (*n* = 3), R1-3 are the different replicates of each site. (**b**) Bar plot on genus level showing years 2013 and 2017 in November at the three different sites (*n* = 3), R1-3 are the different replicates of each site.

**Figure 4:**
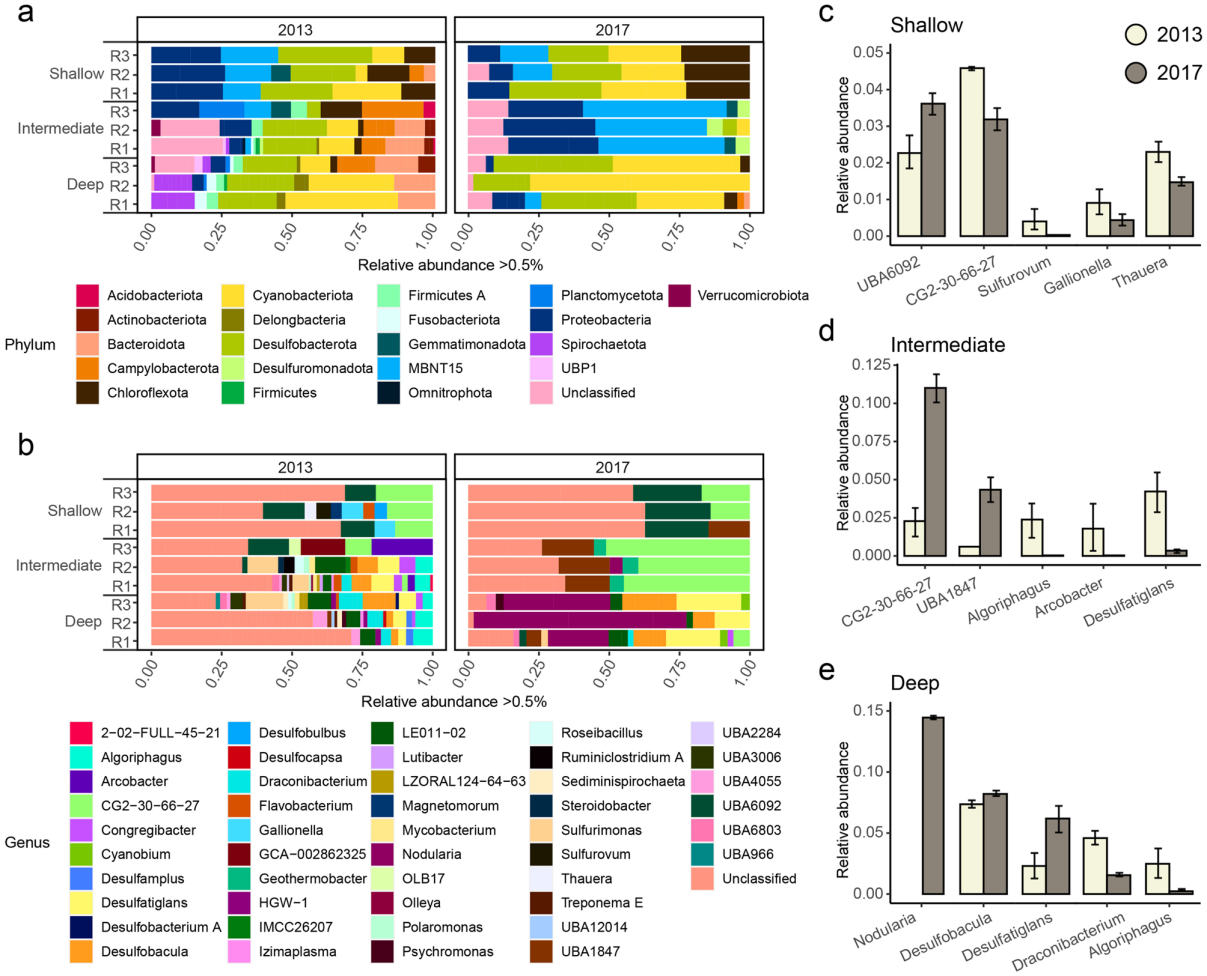
Bar plot of significant differential abundance taxa on phylum and genus level for ASVs over 0.5% relative abundance. The cutoff was based on an ASV having > 0.5% in at least one sample. (**a**) Bar plot on phylum level for 2013 and 2017 in November at the three different sites (*n* = 3), R1-3 are the different replicates of each site. (**b**) Bar plot on genus level shown for 2013 and 2017 in November at the three different sites (*n* = 3), R1-3 are the different replicates of each site. (**c**) Changes in average (*n* = 3) relative abundance of the shallow site on the highest significant changed taxa; shown are year 2013 (beige) and 2017 (dark beige) within five different genera. (**d**) Changes on average (*n* = 3) relative abundance of the intermediate site on the highest significant changed taxa; shown are year 2013 (beige) 2017 (dark beige) within five different genera. (**e**) Changes on average (*n* = 3) relative abundance of the deep site on the highest significant changed taxa; shown are year 2013 (beige) and 2017 (dark beige) within five different genera; Error bars show the mean and standard deviations.

The relative abundance of *Proteobacteria* in the shallow site was higher during 2013 (mean ± SD, 33.2% ± 0.02) when compared to 2017 (24.8% ± 0.01) and had a 6.1% higher relative abundance of *Desulfobacterota* (2013, 11.3% ± 0.002, 2017, 10.6% ± 0.02; Supplemental Fig. S6). Differential abundance analysis on the ASV level showed that 67% of the ASVs differed significantly between 2013 and 2017 (Fig. 3 and Supplemental Table S5 for differential abundance analysis) and that 98.9% of those significantly changed ASVs had a relative abundance below 0.5% each (Fig. 3). Of the 1.1% of significantly different ASVs with a relative abundance over 0.5%, the *Proteobacteria* and *Campylobacterota* decreased while *Chloroflexota* and *Cyanobacteria* increased (Fig. 4, Figs. S7 and Fig. S8 with summarized stacked bars of the replicates plus SD as errors bars, and Supplemental Table S5). In 2017, the number of abundant genera (i.e. above 0.5% relative abundance) decreased that included *CG2-30-66-27* (2013, 4.6% ± 0.01 relative abundance & 2017, 3.1% ± 0.002) belonging to the *MBNT15* phylum, *Sulfurovum* (0.4% ± 0.002 and 0.002% ± 0.0001), *Gallionella* (0.9% ± 0.002 and 0.4% 0.001), and *Thauera* (0.04% ± 0.001 and 0.01% ± 0.001) while genera including *UBA6092* (2.2% ± 0.003 and 3.6% ± 0.003) increased (Fig. 4). SIMPER analysis within the shallow site between the years 2013 and 2017 showed that the average contribution of genera responsible for the differences between communities were rather small compared to the deeper sites, confirming the low significant differential abundant ASVs between the years. Most likely the genera *UBA6092* (3.72% average contribution of dissimilarity), *Caldilinea* (2.56%), and *CG2-30-66-27* (2.47%) contributed to the differences between the years (Supplemental Table S4).

The intermediate site showed a 44% increase in *Proteobacteria* in 2017 (mean ± SD, 27.1% ± 0.01) compared to 2013 (15.1% ± 0.07; Supplemental Fig. S6). Similar to the shallow site, 65.3% of unique ASVs were significantly different between 2013 and 2017 (Fig. 3) while there was a larger proportion (6.7%) of abundant ASVs with > 0.5% relative abundance (Fig. 3 and Supplemental Table S5). Significantly increased phyla within the intermediate site included *MBNT15* and *Proteobacteria* while the most abundant taxa decreased including the *Campylobacterota*, *Bacteroidetes*, *Cyanobacteria*, and *Desulfobacterota* (Fig. 4, Figs. S7 and Fig. S8 with summarized stacked bars of the replicates plus SD as errors bars). A decline of the abundance of significant relative abundant ASVs (> 0.5%) on genera level by approximately 90% was observed from 2013 to 2017, with a significant decrease in e.g. *Algoriphagus* (2013, 2.3% ± 0.009 relative abundance and 2017, 0.03% ± 0.0001), *Arcobacter* (1.7% ± 0.01 and 0.03% ± 0.0001), and *Desulfatiglans* (4.2% ± 0.01 and 0.3% ± 0.001) while uncultured bacteria belonging to e.g. *CG2-30-66-27* (2.2% ± 0.008 and 11.0% ± 0.008) and *UBA1847* (0.6% and 4.3% ± 0.007) genera increased (Fig. 4). The increased relative abundance of these genera were most likely also the main contributor between the differences of the communities from 2013 to 2017 with 11.23% of dissimilarity contribution for *CG2-30-66-27* and 4.78% for *UBA1847* (Supplemental Table S4).

The deep site had the strongest shift in the bacterial community between 2013 and 2017 with an increase of 44% in *Proteobacteria* (mean ± SD, 2013, 8.3% ± 0.03, 2017, 15.1% ± 0.03; Supplemental Fig. S6) that replaced *Desulfobacterota* as the most abundant phylum. Compared to the other two sites, the deepest site had the fewest number of significantly changed ASVs with 63% and concomitantly a larger number of ASVs with a relative abundance of > 0.5% with 11.1% (Fig. 3). Phyla including *Spirochaetota*, *Firmicutes*, and *Bacteroidetes* significantly decreased while *Cyanobacteria* increased in 2017 (Fig. 4, Figs. S7 and Fig. S8 with summarized stacked bars of the replicates plus SD as errors bars). The genus *Nodularia* (2013, not detected and 2017, 14.4% ± 0.01) was most responsible for the significant increase in *Cyanobacteria* in 2017 while the genera *Desulfatiglans* (2.3% ± 0.009 and 6.1% ± 0.009) and *Desulfobacula* (7.3% ± 0.002 and 8.2% ± 0.001) also significantly increased. In contrast, e.g. *Draconibacterium* (4.5% ± 0.004 and 1.5% ± 0.001) and *Algoriphagus* (2.4% ± 0.01 and 0.2% ± 0.001) decreased (Fig. 4). Finally, the diversity of abundant genera significantly decreased from 2013 to 2017 (Fig. 4). These results also confirmed that the main contributors for the differences between the years were most likely the *Nodularia* (20.73% average contribution of dissimilarity) followed by *Desulfobacula* (8.34%) and *Desulfatiglans* (6.21%) (Supplementary Table S4).

## Discussion

Global coastal oceans are continuously influenced by anthropogenic induced changes such as accelerated eutrophication that can result in decreased oxygen concentrations in bottom waters^4^ or the steady influence of ongoing climate change related effects such as rising temperatures that exacerbate existing eutrophication effects^26^. In situ bottom water temperature and oxygen data from March 2017 to December 2017 showed a decline in oxygen concentrations along with a temperature increase in November in coastal bottom waters at all the three sampling sites. These data match that collected by HELCOM and ICES^25^ showing trends of increasing temperatures and declining oxygen concentrations in coastal bottom waters (1–30 mbs) independent of the depth across the Western Gotland-, Eastern Gotland-, and Bornholm Basins (Supplemental Fig. S1). A previous study showed significantly different microbial communities along this coastal water depth gradient in 2013^14^, which could also be shown for the communities of the same gradient in the year 2017. The focus of this study was to investigate Baltic Sea sediment changes between 2013 and 2017 at different coastal water depths to study oxygen deficient zones common in the Baltic Sea^27^.

The deepest investigated coastal sediment (~ 30 mbs) was previously shown to be under consistent oxygen deficient conditions while the area around the bay has a history of long-term oxygen deficiency^14^. However, this area was found to have changed to seasonal varying hypoxic-oxic conditions in 2017 that was potentially due to the reduction of nutrient intake into a close-by bay (~ 4 km) from a nearby sewage-treatment plant^28^. The data suggested that most changes within the bacterial communities happened at the deep oxygen deficient site (11% significant different ASVs comparing 2013 and 2017). *Cyanobacteria*, especially *Nodularia*, went from being undetectable in 2013 to being the most abundant genus in 2017. This increase in *Nodularia* may also have caused the significant increase of organic matter due to deposition of pelagic *Cyanobacteria* on the sediment^29^. A further potential cause of increase in OM could have been due to primary production sources such as green algae or dinoflagellates that bloom in spring^30^ along with Diatoms that bloom in spring^30^ and autumn^31^. This coupled to the generally higher OM content within the deep site may also have been due to decreased OM degradation rates in deeper, less well mixed, and low oxygen conditions^20^. This increase in organic matter likely led to an increase in sulfate reducing bacteria like *Desulfobacula* and *Desulfatiglans* in 2017. Such bacteria have been shown to participate in the mineralization of organic matter^32^, and sulfate reduction can account for as much as up to half of organic matter mineralization^33^. Furthermore, the abundance of sulfate reducers has been shown to be positively correlated with organic nitrogen and phosphorus in the surface layer of sediments^34^, and *Desulfobacula* has been found to be associated with Baltic Sea organic-rich sediments^35^. The higher relative abundance of sulfate reducers may also have caused the significant increase of phosphate as sulfate-reducing bacteria may indirectly contribute to dissolved phosphate release during organic matter mineralization^35^ by the production of sulfide that binds ferric iron and hinders the formation of phosphate binding ferric oxyhydroxides^36^. Another potential contribution to the increased pore water phosphate concentrations in November 2017 was the raised oxygen concentration from March to July 2017 leading to iron oxide (Fe–P) that binds phosphate^37^. As the oxygen concentrations became hypoxic in November, Fe–P dissolution may have increased the release of phosphate into the pore water^38^. The general increase in temperature within the last years could have led to a shift within seasonal changing oxygen states, with possible higher regional river-runoff in spring^39^ feeding even long-term hypoxic deeper coastal sediment with oxygen-rich water. This can lead to greater bacterial metabolism rates, followed by lower oxygen concentrations. Similar results have also been reported from arctic lakes with increasing runoff^40^. The data confirmed that with increasing temperature, an increase of cyanobacterial deposition in already hypoxic sediments is likely expected^41^. In addition, more organic matter leads to an increased sulfur cycling close to the sediment surface^34,42^, that might accelerate oxygen decline with subsequent increased release of phosphates^43^. Due to prolonged seasonal stagnant water with less mixing of the bottom water, deeper sites are more affected by eutrophication and can turn long-term hypoxic^44^, and an increase in temperature is likely to accelerate eutrophication induced hypoxia of benthic waters.

In comparison, intermediate coastal sediments (intermediate site, ~ 20 mbs) exposed to already long-term fluctuating hypoxic-oxic conditions may already have had a bacterial community that was adapted to oxygen fluctuations. Compared to 2013 less dominant bacteria taxa were found in 2017. These few dominant taxa include *CG2-30-66-27* from the candidate phylum *MBNT15*, which might be obligate anaerobes that couple H_2_ and acetate oxidation to nitrate reduction^45^. The genus *UBA1847* from the family *Woeseiaceae* within the *Proteobacteria* that are among the most abundant microorganism in coastal sediments. They cover a broad spectrum ranging from facultative sulfur- and hydrogen-based chemolithoautotrophic to obligate chemoorganoheterotrophic bacteria^46^ (Fig. 4). The significantly increased relative abundance of ASVs aligning within the candidate phylum *MBNT15* that thrives in anoxic deep sediments^45^ could be the result of several factors. Either an environmental variation between 2013 and 2017 occurred or the proportion of anoxic sediment within the sliced 0–1 cm increased compared to 2013. In general, the data based on environmental variables in 2017 could suggest a potential reversal of eutrophication effects when compared to 2013, with for example an increase in oxygen concentrations and a decline in phosphorus^23^. In addition to the minor changes in the microbial communities, the data suggested that oxygen histories may play an important role in the extent microbial communities react to environmental changes. This importance was already shown in previous studies, where long-term anoxic coastal Baltic Sea sediment has been re-oxygenated^14^.

Shallow coastal areas are in general more exposed to anthropogenic influenced changes related to climate change or eutrophication, such as input of nutrients from land^47^, increased acidification^48^, and faster warming^49^ of the water column reaching the sediments. However, these sediments have due to their nature a constant oxygen input from e.g. mixing of the water column by the wind and previous studies show that the shallow area has been under long-term oxic conditions^14^. Despite that the studied coastal bay has not undergone any artificial changes, beside decreased nutrient intake of a wastewater treatment plant of a close-by bay (~ 4 km) in the intervening years^28^, seasonally changing oxygen conditions were observed between March and December 2017 (Supplemental Fig. S2). In addition, a previous study shows significant differences between bacterial communities at all three sites with the shallow site being most distinct from the other two^14^. Within the shallow site, only 1.1% of the dominant taxa (i.e. comprising > 0.5% relative abundance) were significantly different comparing 2013 and 2017 indicating the highly abundant bacteria were still present (Fig. 3). Sinkko et al. ^32^ showed that the most abundant ASVs in shallow coastal sediments (~ 4 mbs) are also the most stable fraction of the community during oxygen deficiency events. This indicated that a large part of this community were likely facultative anaerobes. Members of this group include the phyla *Planctomycetota* and *Proteobacteria* that are adapted to temporally varying oxygen concentrations and short-term low oxygen events. The data on one hand showed only minor changes on the bacterial community of the shallow coastal sediments within the Baltic Sea basin in 2017 compared to 2013. Changes in environmental factors such as temperature, pH, oxygen, and organic matter could be either the result of temporal differences or be a first hint of a changing environment. The environmental conversion could be related to climate change with temperature increase, acidification^50^, organic matter accumulation leading to CO_2_ release via decomposition^51^ followed by decreasing oxygen concentration. On the other hand, the intermediate site, already adapted to seasonal oxygen changes, could either show temporal differences over the time or could have potentially showed first hints of a reversed eutrophication. Nevertheless, shallow coastal ecosystems and their bacterial communities will be directly affected by environmental changes related to future climate change effects in combination with already increased nutrient load from the land, and how bacterial communities will adapt to changes needs to be further investigated.

## Conclusions

No simple, single response within bacterial communities of changes within coastal sediments was discerned and how bacterial communities react to environmental changes like eutrophication and/or climate change are an interplay of different spatiotemporal factors including water depth and oxygen supply. Sediments exposed to long-term hypoxic conditions were sensitive to even small changes in e.g. oxygen concentration and temperature increase, probably resulting in accelerated bacterial activity in combination with increased organic matter leading to potential future expansion of already existing hypoxia zones. Bacterial communities of oxygen rich shallow coastal sediments did not show strong responses to environmental changes, showing that these communities were most likely stable under potential transitory oxygen depletion. Continuous exposure to eutrophication, warming, and other climate change related effects has the potential to expand and aggravate already existing hypoxic zones.

## Materials and methods

### Study site

The sampling location was a coastal Baltic Sea bay near the town of Loftahammaer, Sweden. The three sampling sites (Supplemental Fig. S9 and Supplemental Table S2) have different oxygenation histories and were designated as ‘shallow’ (6.5 mbs; WGS 57 53.214, 16 35.934), ‘intermediate’ (20.5 mbs; WGS 57 53.545, 16 35.476), and ‘deep’ (30.1 mbs; WGS 57 53.531, 16 35.165). Detailed descriptions of the sediments are published in previous studies^14,23,24,52^.

### Sampling

Sampling was conducted at each site on 2 November 2017 (*n* = 3). In addition, published data^14^ from the same sites (also *n* = 3 per site) from 2013 were used for comparison^14^. Sampling was conducted as previously described^14^ using acrylic cores (internal diameter 7 cm, length 60 cm) to sample the sediment surface with a Kajak gravity corer at each location. Oxygen and temperature were measured in situ (Multiline™ sensor, WTW™) in the bottom waters (~ 10 cm above sediment; Supplemental Table S2). The sediment surface (0–1 cm) was sliced and transferred into a sterile 50 mL polypropylene centrifuge tube (Thermo Scientific™) and mixed until homogenized. A subsample of 15 mL homogenized sediment was transferred into an acid washed 15 mL polypropylene centrifuge tube (Thermo Scientific™) for pore water analysis as well as an additional subsample of 2 mL for organic matter analysis. The sediment remaining in the initial tube was used for DNA extraction. Samples for chemistry analysis and DNA extraction were stored cooled until transported back to the laboratory the same day and then stored until further analysis at − 20 °C and − 80 °C, respectively^14^. Additionally, oxygen data were measured on bottom water in situ from March 2017 to December 2017 (*n* = 9) on all three sites (Supplemental Fig. S2, Table S2).

### Chemistry analysis

Chemistry analyses were conducted on pore waters and the sediment itself. Pore waters were sampled by centrifuging the 15 mL sediment tubes at 2200 × *g* for 15 min and the supernatant transferred into a new 15 mL acid washed centrifuge tube (Thermo Scientific™). The pore water was filtered through a 0.7 µm Target2™ GMF Syringe Filter (Thermo Scientific™) and pH was measured with a pH electrode (pHenomenal, VWR™). Sulfate, dissolved inorganic phosphate, total iron, nitrate + nitrite (in combination), and organic matter were measured as previously described^14^ except for organic matter samples that were dried for a total of six days at 40 °C instead of 3 days at 80 °C.

### DNA extraction

For extraction of sediment DNA, the samples were homogenized and the DNeasy^®^ PowerSoil Extraction Kit (QIAGEN) was used according to the manufacturer’s guidelines. The extracted DNA was measured using Qubit^®^ 2.0 (Invitrogen™, Life Technologies Corporation) and stored at − 20 °C until 16S rRNA gene amplification and Illumina library preparation. DNA was amplified with 16S rRNA gene PCR primers 341f and 805r^53^ (mainly targeting Bacteria) using a modified PCR program and libraries prepared with the Illumina Nextera kit according to Lindh et al.^54^.

### Sequencing and bioinformatics analysis

The DNA samples were sequenced at the Science for Life Laboratory (SciLifeLab) in Stockholm on the MiSeq platform with 2 × 301 bp pair-end setup. The sequences were analyzed using the DADA2 pipeline^55^ (https://benjjneb.github.io/dada2/index.html) on the UPPMAX cluster (Uppsala Multidisciplinary Center for Advanced Computational Science). Parameters for the quality trimming of the sequences were at 290 (forward read) and 230 (reverse read) with left trimmed at 21 bp. The error model was run with a MAX_CONSIST of 30 for reads, and the R1 and R2 pairs were merged with minimum overlap of 10 bp and zero mismatch allowance. The average sequence count after quality trimming was 144,259 (min. 9330 and max. 428,077; Supplemental Table S6). Chimeras were removed and the taxonomy was assigned against the Genome Taxonomy Database^56^ (GTDB version 86) using default parameters with DADA2, before the final data were analyzed using R version 3.5.2^57^.

### Statistical approaches

To determine the sequencing depth, rarefaction curves were calculated with the ‘vegan´ package version 2.5-6^54^ based on the number of ASVs and final read counts after DADA2 analysis. The dataset showed differences in sequencing depth between the years. To confirm our results based on scaling with ranked subsampling and relative abundance normalization, the data have been compared to results using rarefied samples that showed the methods were suitable (Supplemental Table S3).

Diversity of the microbial communities was estimated by calculating the alpha diversity (Shannon’s H index) based on counts normalized using scaling with ranked subsampling^55^. To test for significant differences in diversity between years and sites a linear regression model was used due to the nested nature of the data with three replicates per site and the interaction between the sites and years. Different models were fitted and the model that showed the best fit for the data structure was chosen based on Akaike information criterion (AIC). All factors (years and sampling sites as interaction and replicates nested within sites) were used as fixed effects in the final model (‘lme()’ function, ‘stats’ package)^57^ due to the small sample size. The statistical differences between the factors were tested using the ‘anova’ function from the ‘stats’ package in R^53^ with pairwise comparison for years at each site using the ‘emmeans’ package (version 1.5.4)^56^. For testing on differential abundances between the years at each sampling site, taxa not seen in at least 20% of the samples were removed and a zero-inflated negative binominal model was used to reduce the excess of zeros in the dataset. Then, differential abundance analysis on counts was done with the ‘DESeq2´^57^ package to analyze statistical differences between communities based on all ASVs. The cutoff of 0.5% was based on an ASV having > 0.5% relative abundance in at least one sample. For further analysis (if not otherwise stated), relative abundances were calculated from all ASV count data. Beta-diversity was investigated via non-metric multidimensional scaling (NMDS) based on Bray–Curtis distances. Variance inflation factor analysis showed multi-collinearity between oxygen and nitrate (Supplemental Table S1). The focus of this study was to investigate changes based on oxygen and temperature. Therefore, the variable nitrate has been removed for the redundancy analysis (RDA), which resulted in VIF < 5 for the remaining variables. The RDA analysis was performed using the ‘RDA()’ function within the ‘vegan’ package plus an ANOVA permutation test, with a direct model using marginal terms to investigate if significant environmental variables influenced the differences between the bacterial communities. Bray–Curtis dissimilarities were calculated using the ‘vegdist’ function within the ‘vegan’ package. To check if microbial community composition in 2013 and 2017 was significantly different, a PERMANOVA within the ‘adonis’ function in the ‘vegan’ package with Benjamin–Hochberg p-value correction was applied. Additionally, PERMANOVA testing was used to compare the microbial communities along the depth gradient (comparing the different sites) for the years 2013 and 2017. Similarity of percentages (SIMPER) analysis from the `vegan’ package was used to discriminate species responsible for dissimilarities between the year 2013 and 2017 at each site, as well as between sites within each year (Supplemental Table S4). To determine whether differences in environmental variables between the years were significant, the same model that was used for diversity testing was constructed and tested. The best fit (best AIC) for the data structure was reached using a linear regression model (‘lme()’ function, ‘stats’ package) with years and sampling sites as interaction and replicate nested within site as fixed effects.

## Supplementary Information

Supplementary Information 1.

Supplementary Information 2.

Supplementary Information 3.

Supplementary Information 4.

Supplementary Information 5.

## Data Availability

16S rRNA gene sequencing data are available on the NCBI database under Bioproject PRJNA721576 and PRJNA322450.

## Code availability

The code used is available at Supplemental Methods S1.
