# Supplementary material for: Interplay between eutrophication and climate warming on bacterial communities in coastal sediments differs depending on water depth and oxygen history
Source: Sci Rep. 2021 Dec 3;11:23384. doi: 10.1038/s41598-021-02725-x (PMC8642432; doi:10.1038/s41598-021-02725-x)
Supplement: Supplementary file 1 — Supplementary Information 1. [file 41598_2021_2725_MOESM1_ESM.pdf]

Supplementary information

Historical information of the studied area.

Data of temperature, oxygen, phosphate, nitrate, and nitrite from the nearby observation station from 2001-2012 are available by the Kalmar county coastal water committee at (Swedish website):

http://www.kalmarlanskustvatten.org/index.php?option=com_herbfileselector&view=herbfileselector&Itemid=3.

Methods S1 | Code used. The code used to process the 16S rRNA gene amplicon and chemistry data.

Table S1 | Statistical tests. Statistical approaches used for comparing geochemical data, diversity indices, and community composition between 2013 and 2017 in the shallow, intermediate, and deep sites.

Table S2 | Sample information. Metadata of triplicate samples from the years 2013 and 2017 for each site. Included are information about sampling location, time of sampling, and environmental variables as well as information of the oxygen concentration from March 2017 to December 2017 for each sampling site. Information about relative abundance of each amplicon sequence variant (ASV) and taxonomical annotation of each sample is listed.

Table S3 | Rarefied data. Analysis of Shannon´s H Diversity, NMDS, and bar plots on phylum and genus levels using rarefied data. The data are rarefied based on the smallest sample size *n*=1564.

Table S4 | Similarity of percentages analysis (SIMPER). Analysis of contribution of dissimilarity between bacterial communities using SIMPER on genus level. Shown are the community contributor on genus level; the average contribution to overall dissimilarity; standard deviation of contribution; ratio of average to standard deviation of contribution; average of abundances in each treatment (a & b) and the cumulative contributions. SIMPER was calculated for each site (shallow, intermediate & deep) comparing the years 2013 and 2017, as well as comparing the sites between each other in 2013 and 2017.

Table S5 | Differential abundance of 16S rRNA gene amplicon data. Differential abundance analysis of all three sites comparing the years 2013 and 2017 on the 16S rRNA gene amplicon dataset. Analysis was performed with the package DESeq2 using counts summarized on genus level. Low abundant taxa that were not seen in at least 20 % of the samples were filtered out. Zero inflated count model was used to reduce zeros in the counts matrix and therefore, to increase statistical power of the analysis; Genus, baseMEAN, e.g. shallow2013 = baseMean of shallow site in 2013, shallow2017 = baseMean of shallow site in 2017; log2FoldChange; lfcSE= LogFoldChange Standard Error; stats; p-value; padj = corrected p-value with Benjamin Hochberg correction.

Table S6 | Sequencing information. Information on the sequencing and quality control of the 16S rRNA gene amplicon analysis including read length during sequencing, read length in million reads per sample, amount of sequences introduced in the dada2 pipeline before and after quality trimming and the filter step, as well as the number of ASVs after chimera removal.

Figure S1 | Baltic Sea trends from 2000-2019 by ICES and HELCOM. Trends of temperature and oxygen parameters measured in November between 2000 and 2019 within several observational stations held by ICES and HELCOM across the Baltic Sea (area between Western Gotland Basin, Eastern Gotland Basin, and Bornholm Basin) and coastal depth (1-30 mbs). (a) Temperature (°C) graph shown data in month November between 2000 and 2019 (x-axis) across the Baltic Sea of depth between 1 and 30 m; linear trend line of data is shown in blue. Regression line equation as well as R-square are shown within the plot. (b) Oxygen (mg/L) graph shown data in month November between 2000 and 2019 (x-axis) across the Baltic Sea of depth between 1 and 30 m; linear trend line of data is shown in blue. Regression line equation as well as R-square are shown within the plot. Data was collected and provided by HELCOM within the International Council for the Exploration of the Sea (ICES), and can be found on: data.ices.dk/view-map.

Figure S2 | Oxygen concentration monitored in 2017. Measured oxygen concentration in mg/L (y-axis) in bottom waters of each sampling site (n=1) between March and December 2017 (n=9) (x-axis). Sampling sites are colored: shallow (salmon), intermediate (green), and deep (black)) with the hypoxic border of 2 mg/L as a light grey dotted line. No data are available for June and for the deep site in October.

Figure S3 | Rarefaction curve and Shannon Diversity Index. (a) Rarefaction curve of the analyzed samples for the shallow (salmon), intermediate (green), and deep (black) sites in 2013 and 2017 where the minimum sample count achieved was 1564; (b) Shannon diversity index on amplicon sequence variants (ASVs) using 16S rRNA gene amplicon sequencing data. Data were normalized using scaling with ranked subsampling[54]. Boxplots of Shannon Index calculated for the shallow (*n=3*), intermediate (*n=3*), and deep (*n=3*) sites in 2013 and 2017; significant p-values, **: $p<0.01$ *: $p<0.05$.

Figure S4 | Bray-Curtis dissimilarity heat map. Pairwise comparison of bacterial community composition of each sample based on Bray-Curtis dissimilarities. The highest dissimilarity is indicated with 1.00 (purple), more similar samples are indicated with 0.50 (green), and identical samples with 0.00 (yellow). Each year and site combination were compared with each other with R1-R3 indicating the replicates of each site.

Figure S5 | Redundancy Analysis (RDA). Overview of the constrained ordination analysis of the different sampling sites (shallow (salmon), intermediate (green), and deep (black)) within the two selected years (*n*=15) in dependence of the examined environmental variables. Environmental variables with significant influence on the bacterial communities are shown with an asterisk.

Figure S6 | Bar plot of relative abundance on phylum level. Overview of ASVs with a relative abundance > 0.5 % summarized on phylum level per sampling site (*n*=3) and year (*n*=2) within the month November.

Figure S7 | Bar plot of relative abundance on phylum and genus levels. Bar plots of significantly differential abundant taxa over 0.5 % relative abundance based on ASVs and annotated genus level; the cutoff was based on an ASV having >0.5 % in at least one sample; summarized are phyla <0.5 % and non-significant phyla > 0.5 %. Shown are the average relative abundance of the three replicates with standard deviation (error bars) where ASVs have been at least *n*=3. (a) The plot on phylum level showing years 2013 and 2017 in November at the three different sites (*n*=3). (b) Bar plot on genus level showing years 2013 and 2017 in November at the three different sites (*n*=3).

Figure S8 | Bar plot of significant differential abundance taxa on phylum and genus level for ASVs over 0.5 % relative abundance. The cutoff was based on an ASV having >0.5 % in at least one sample. Shown are the average relative abundance of the three replicates with standard deviation (error bars) where ASVs have been at least *n*=3. (a) Bar plot on phylum level for 2013 and 2017 in November at the three different sites (*n*=3). (b) Bar plot on genus level shown for 2013 and 2017 in November at the three different sites (*n*=3).

Figure S9 | Sampling site map. Overview about the selected sampling sites from 2013 and 2017 close to the town of Loftahammaer, Sweden; shown are the shallow (6.5 m), intermediate (20.5 m), and deep (30.1 m) site.

Supplemental Figures

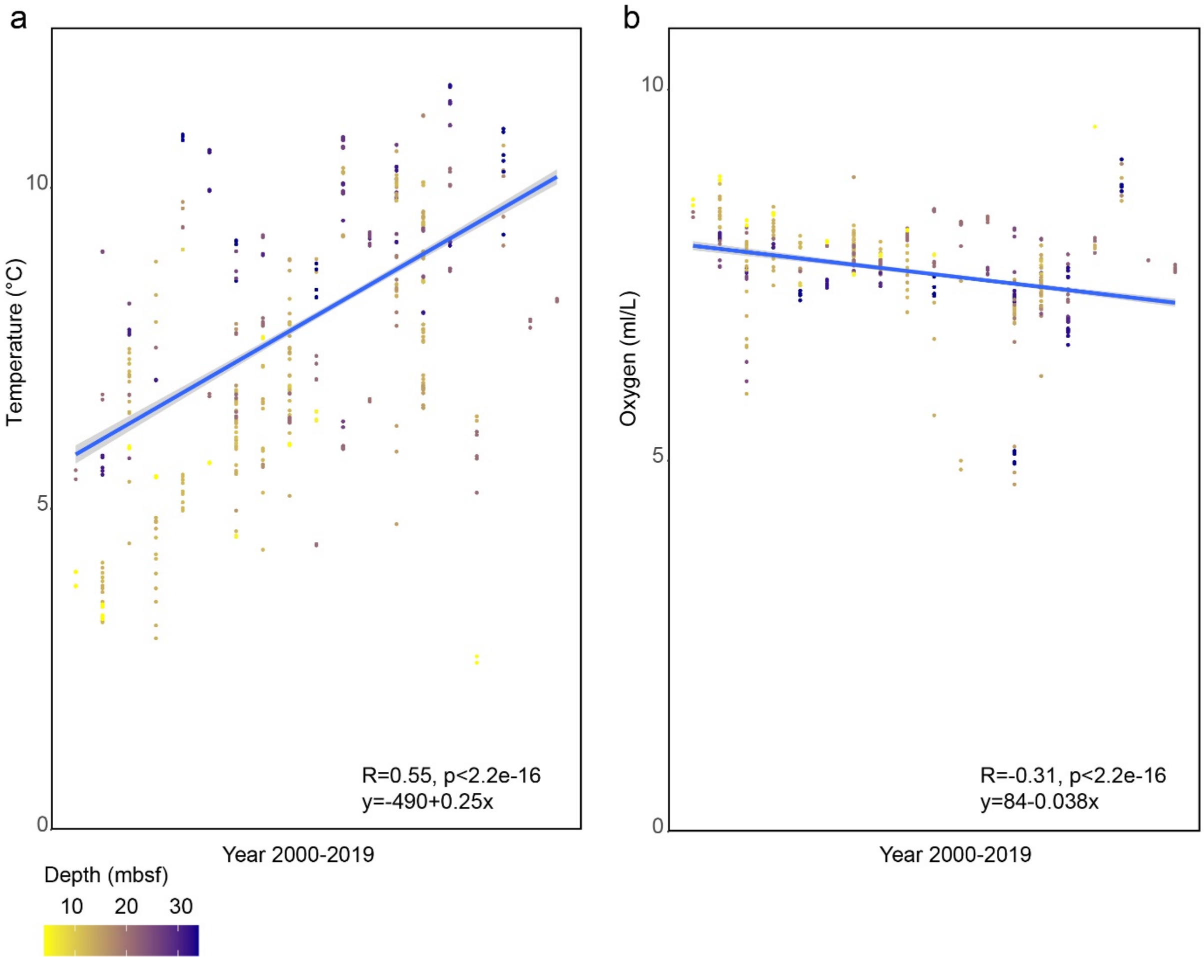

Figure S1

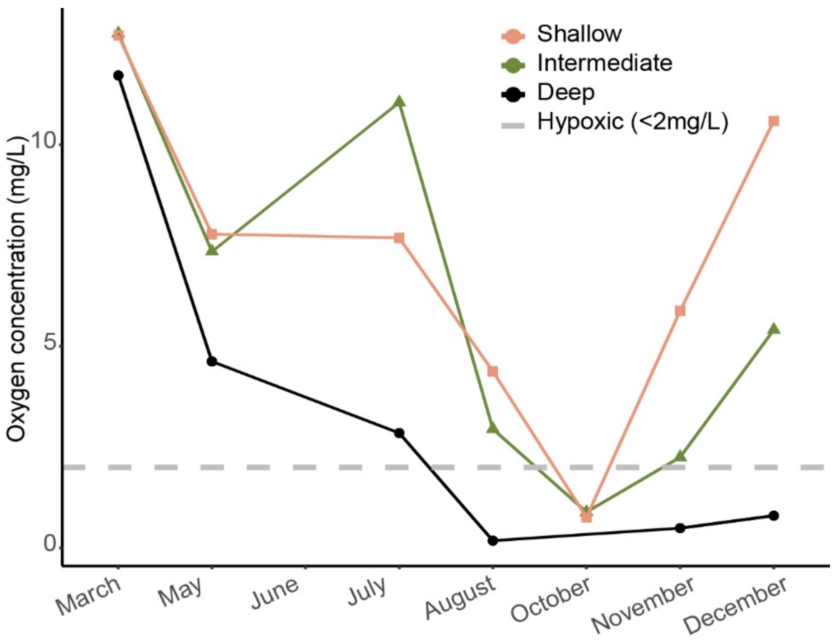

Shallow
Intermediate
Deep
Hypoxic (<2mg/L)
Oxygen concentration (mg/L)
10
5
0
March
May
June
July
August
October
November
December

Figure S2

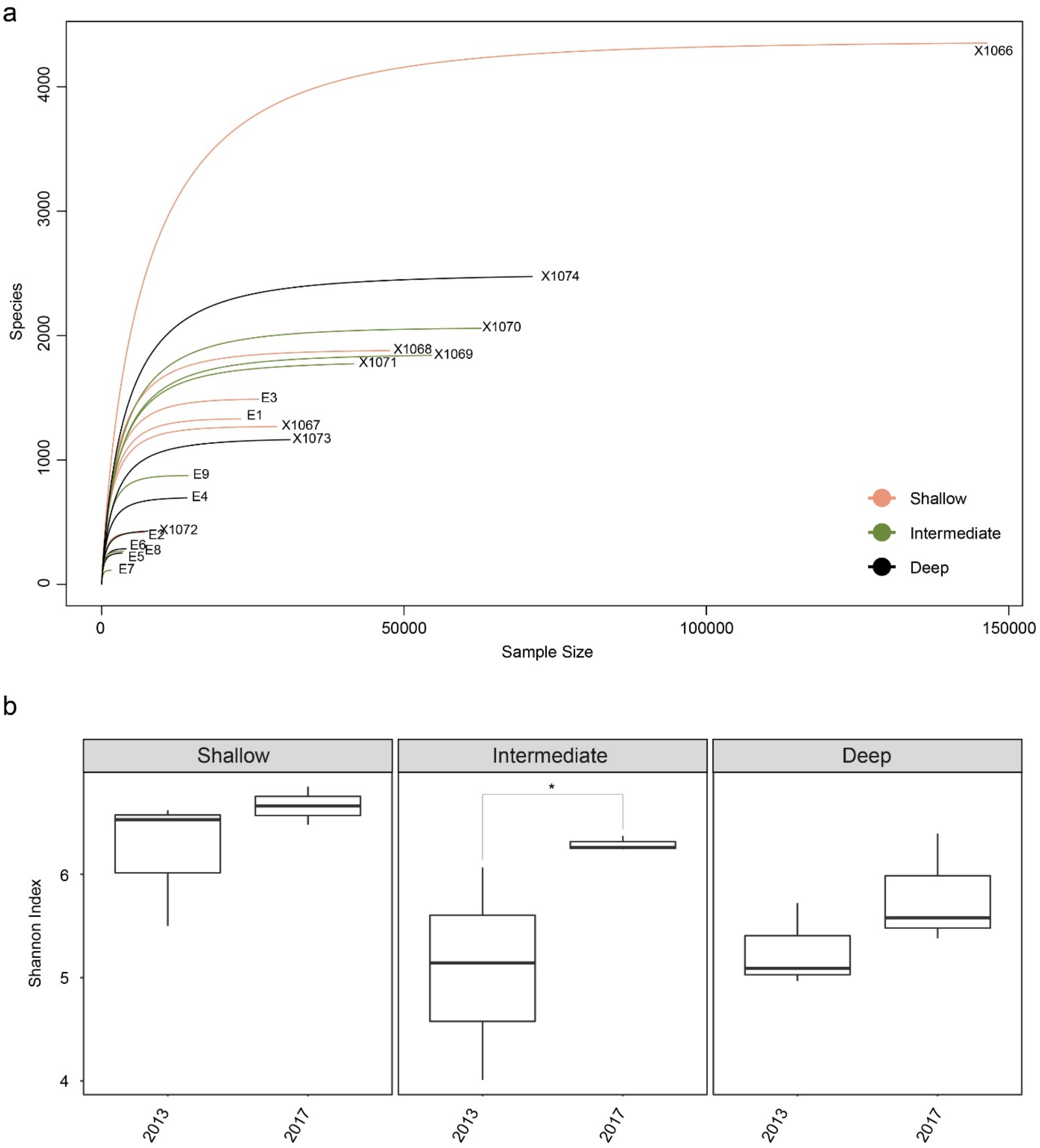
a
Species
Sample Size
0
1000
2000
3000
4000
0
50000
100000
150000
X1066
X1074
X1070
X1068
X1069
X1071
E3
E1
X1067
X1073
E9
E4
E2
X1072
E6
E8
E5
E7
Shallow
Intermediate
Deep
b
Shallow
Intermediate
Deep
Shannon Index
4
5
6
*
2013
2017
2013
2017
2013
2017

Figure S3

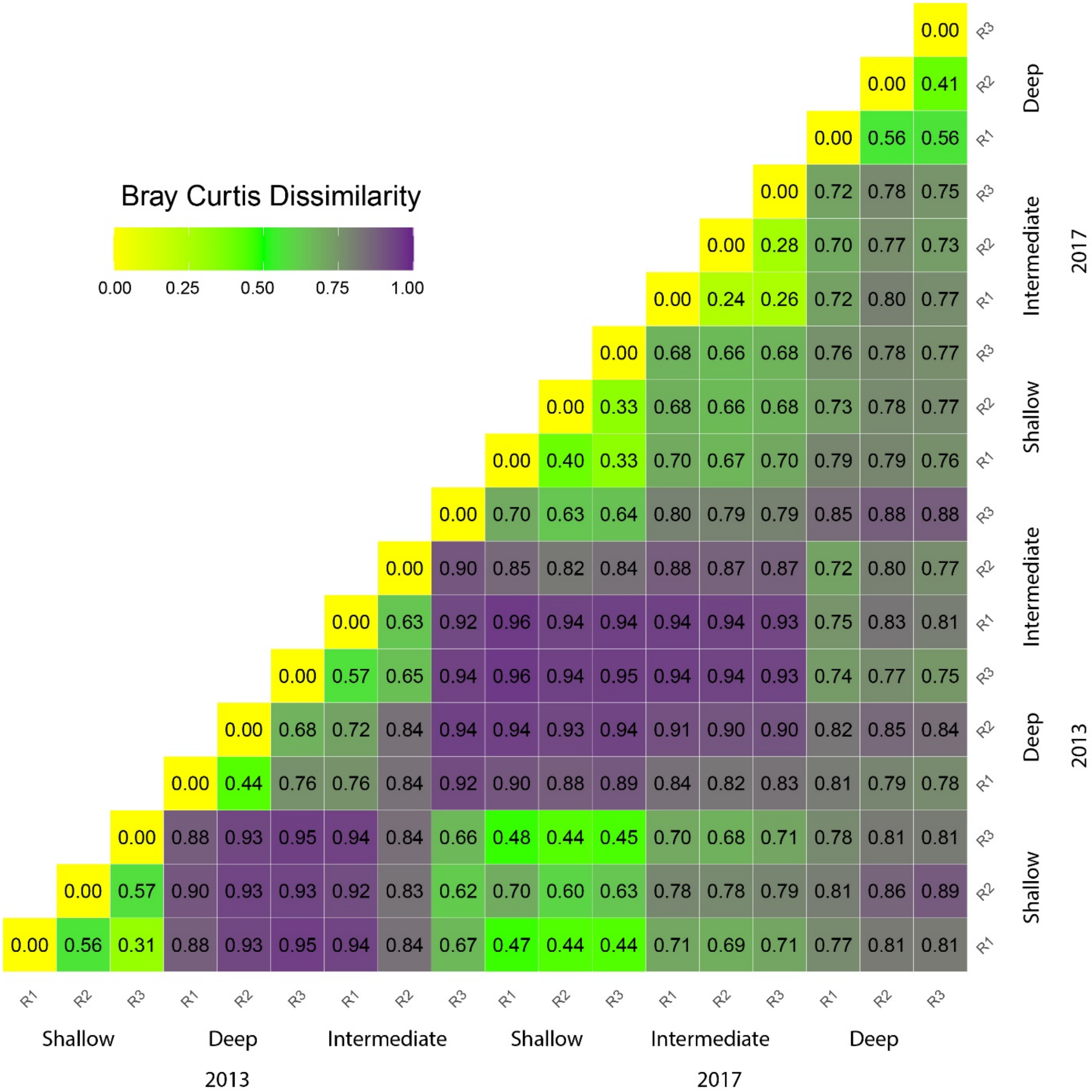
Bray Curtis Dissimilarity
0.00 0.25 0.50 0.75 1.00
0.00
0.00 0.41
0.00 0.56 0.56
0.00 0.72 0.78 0.75
0.00 0.28 0.70 0.77 0.73
0.00 0.24 0.26 0.72 0.80 0.77
0.00 0.68 0.66 0.68 0.76 0.78 0.77
0.00 0.33 0.68 0.66 0.68 0.73 0.78 0.77
0.00 0.40 0.33 0.70 0.67 0.70 0.79 0.79 0.76
0.00 0.70 0.63 0.64 0.80 0.79 0.79 0.85 0.88 0.88
0.00 0.90 0.85 0.82 0.84 0.88 0.87 0.87 0.72 0.80 0.77
0.00 0.63 0.92 0.96 0.94 0.94 0.94 0.94 0.93 0.75 0.83 0.81
0.00 0.57 0.65 0.94 0.96 0.94 0.95 0.94 0.94 0.93 0.74 0.77 0.75
0.00 0.68 0.72 0.84 0.94 0.94 0.93 0.94 0.91 0.90 0.90 0.82 0.85 0.84
0.00 0.44 0.76 0.76 0.84 0.92 0.90 0.88 0.89 0.84 0.82 0.83 0.81 0.79 0.78
0.00 0.88 0.93 0.95 0.94 0.84 0.66 0.48 0.44 0.45 0.70 0.68 0.71 0.78 0.81 0.81
0.00 0.57 0.90 0.93 0.93 0.92 0.83 0.62 0.70 0.60 0.63 0.78 0.78 0.79 0.81 0.86 0.89
0.00 0.56 0.31 0.88 0.93 0.95 0.94 0.84 0.67 0.47 0.44 0.44 0.71 0.69 0.71 0.77 0.81 0.81
R1 R2 R3 R1 R2 R3 R1 R2 R3 R1 R2 R3 R1 R2 R3 R1 R2 R3
Shallow Deep Intermediate Shallow Intermediate Deep
2013 2017
Deep
Intermediate
Shallow
Intermediate
Deep
Shallow
2017
2013

Figure S4

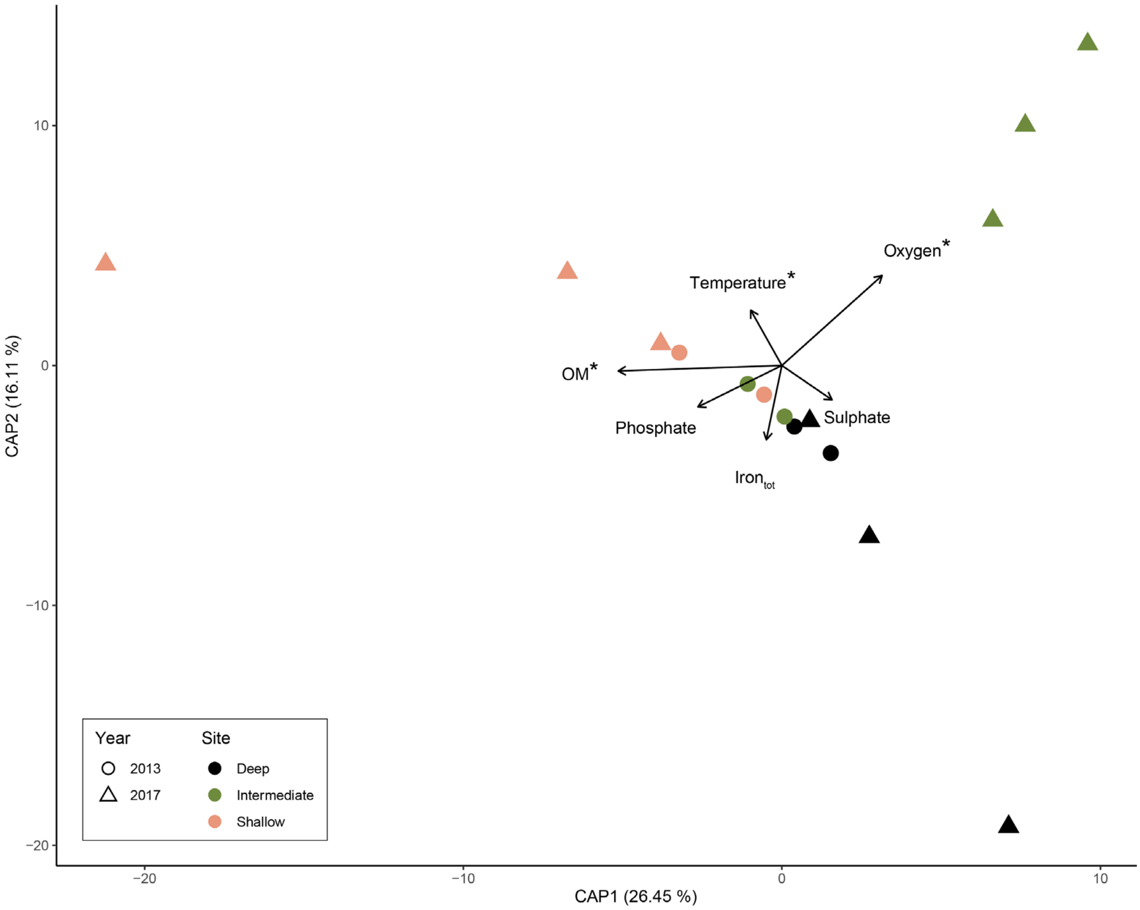

CAP2 (16.11 %)
10
0
−10
−20
−20
−10
0
10
CAP1 (26.45 %)
Oxygen*
Temperature*
OM*
Phosphate
Sulphate
Iron$_{tot}$
Year
2013
2017
Site
Deep
Intermediate
Shallow

Figure S5

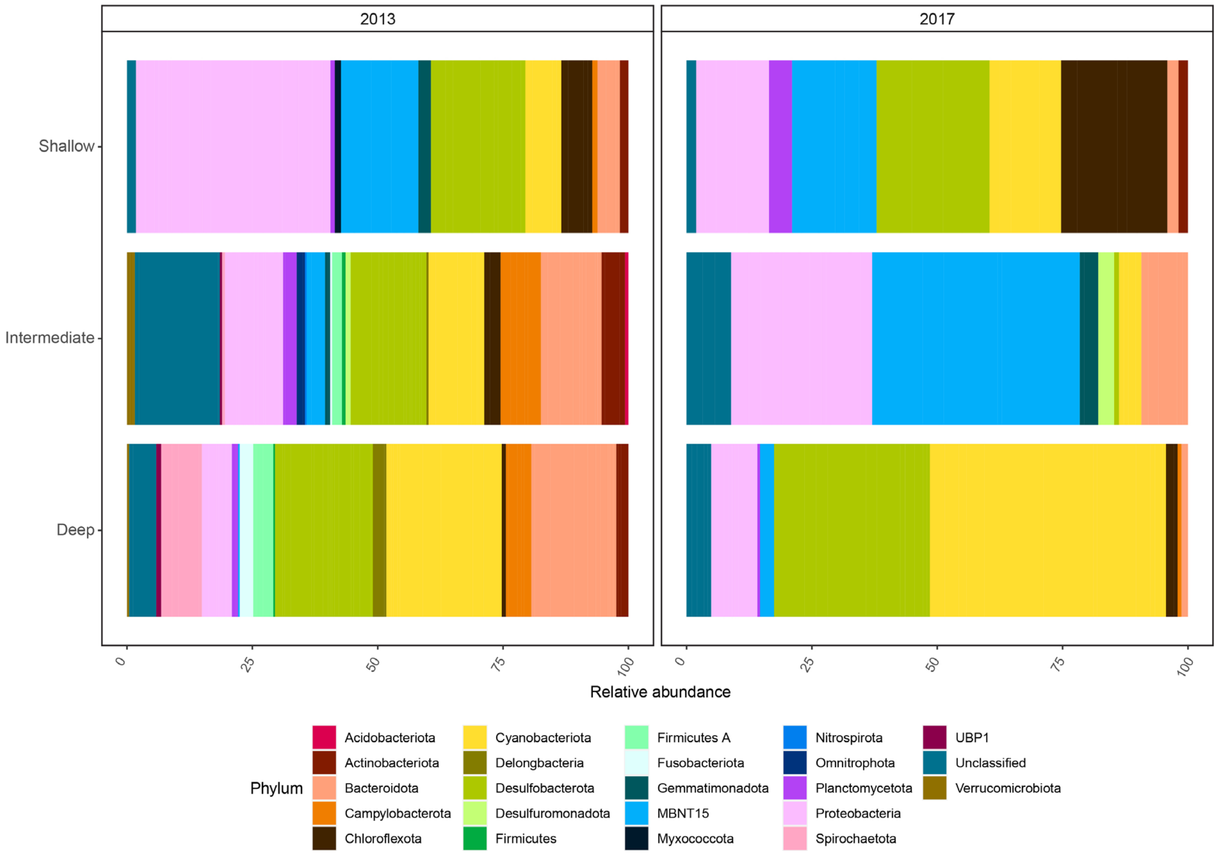
2013
2017
Shallow
Intermediate
Deep
0
25
50
75
100
Relative abundance
Phylum
Acidobacteriota
Actinobacteriota
Bacteroidota
Campylobacterota
Chloroflexota
Cyanobacteriota
Delongbacteria
Desulfobacterota
Desulfuromonadota
Firmicutes
Firmicutes A
Fusobacteriota
Gemmatimonadota
MBNT15
Myxococcota
Nitrospirota
Omnitrophota
Planctomycetota
Proteobacteria
Spirochaetota
UBP1
Unclassified
Verrucomicrobiota

Figure S6

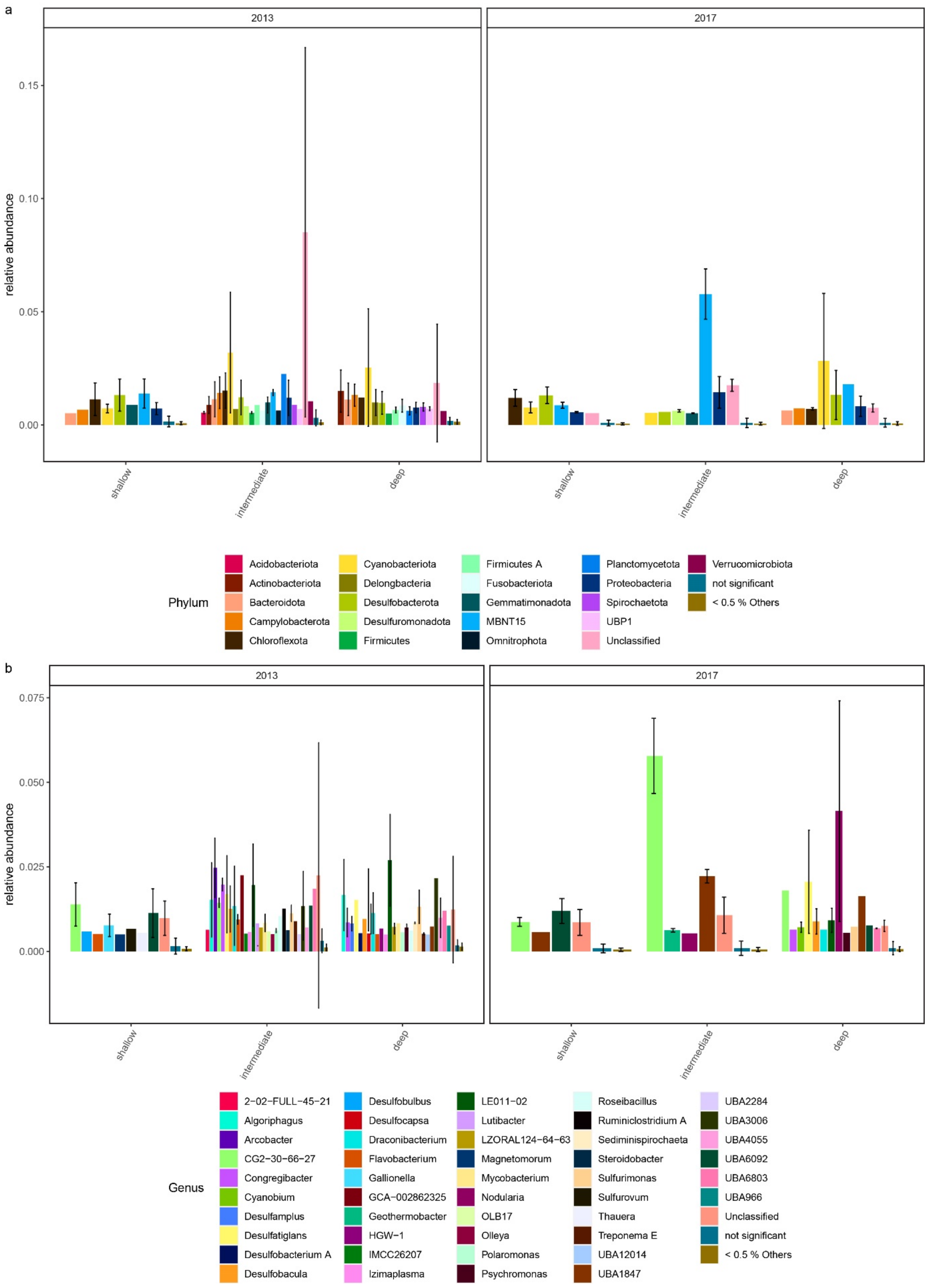
a
2013
2017
relative abundance
0.15
0.10
0.05
0.00
shallow
intermediate
deep
Phylum
Acidobacteriota
Actinobacteriota
Bacteroidota
Campylobacterota
Chloroflexota
Cyanobacteriota
Delongbacteria
Desulfobacterota
Desulfuromonadota
Firmicutes
Firmicutes A
Fusobacteriota
Gemmatimonadota
MBNT15
Omnitrophota
Planctomycetota
Proteobacteria
Spirochaetota
UBP1
Unclassified
Verrucomicrobiota
not significant
< 0.5 % Others
b
2013
2017
relative abundance
0.075
0.050
0.025
0.000
shallow
intermediate
deep
Genus
2−02−FULL−45−21
Algoriphagus
Arcobacter
CG2−30−66−27
Congregibacter
Cyanobium
Desulfamplus
Desulfatiglans
Desulfobacterium A
Desulfobacula
Desulfobulbus
Desulfocapsa
Draconibacterium
Flavobacterium
Gallionella
GCA−002862325
Geothermobacter
HGW−1
IMCC26207
Izimaplasma
LE011−02
Lutibacter
LZORAL124−64−63
Magnetomorum
Mycobacterium
Nodularia
OLB17
Olleya
Polaromonas
Psychromonas
Roseibacillus
Ruminiclostridium A
Sediminispirochaeta
Steroidobacter
Sulfurimonas
Sulfurovum
Thauera
Treponema E
UBA12014
UBA1847
UBA2284
UBA3006
UBA4055
UBA6092
UBA6803
UBA966
Unclassified
not significant
< 0.5 % Others

Figure S7

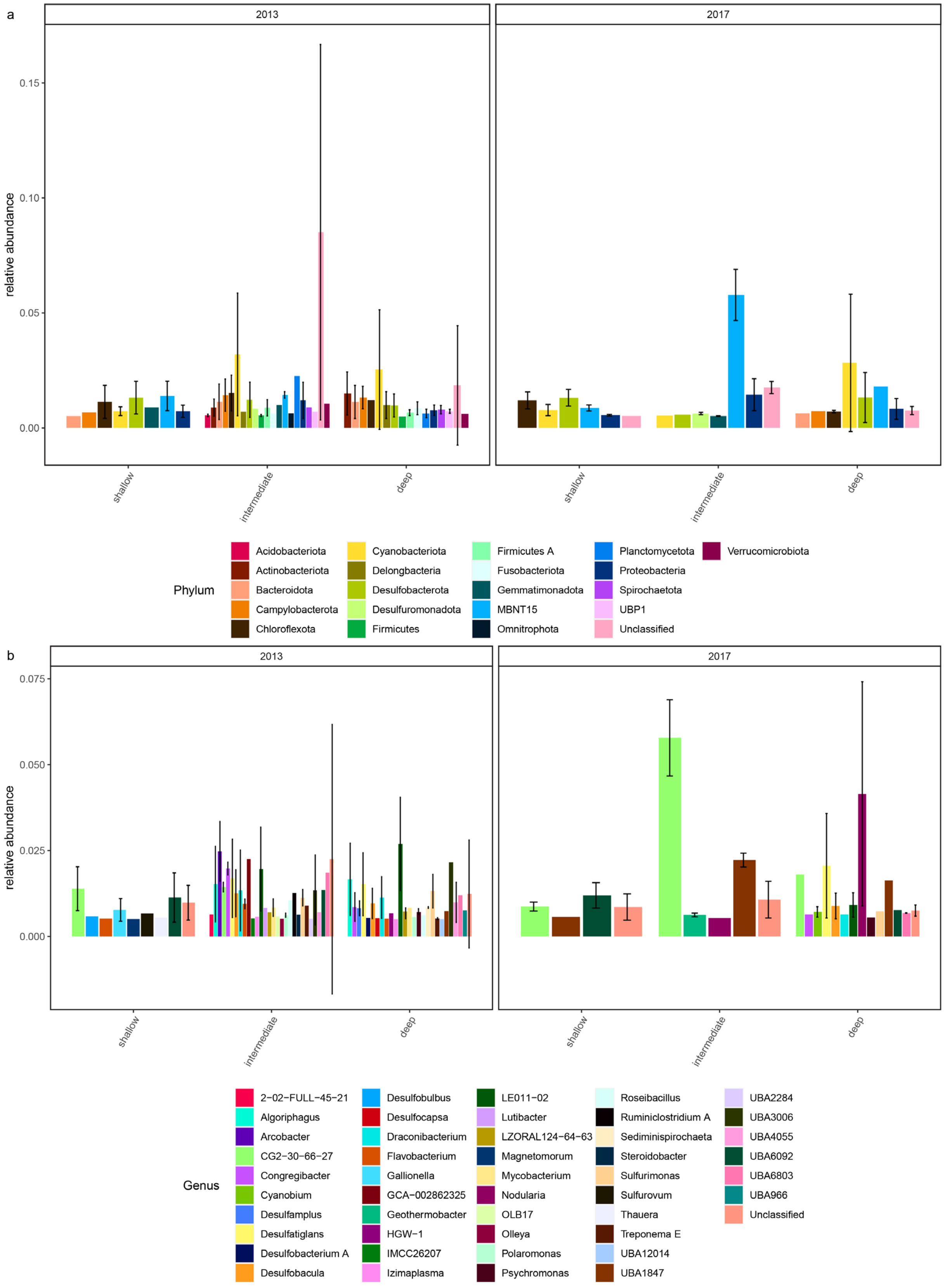
a
2013
2017
relative abundance
0.15
0.10
0.05
0.00
shallow
intermediate
deep
Phylum
Acidobacteriota
Actinobacteriota
Bacteroidota
Campylobacterota
Chloroflexota
Cyanobacteriota
Delongbacteria
Desulfobacterota
Desulfuromonadota
Firmicutes
Firmicutes A
Fusobacteriota
Gemmatimonadota
MBNT15
Omnitrophota
Planctomycetota
Proteobacteria
Spirochaetota
UBP1
Unclassified
Verrucomicrobiota
b
2013
2017
relative abundance
0.075
0.050
0.025
0.000
shallow
intermediate
deep
Genus
2−02−FULL−45−21
Algoriphagus
Arcobacter
CG2−30−66−27
Congregibacter
Cyanobium
Desulfamplus
Desulfatiglans
Desulfobacterium A
Desulfobacula
Desulfobulbus
Desulfocapsa
Draconibacterium
Flavobacterium
Gallionella
GCA−002862325
Geothermobacter
HGW−1
IMCC26207
Izimaplasma
LE011−02
Lutibacter
LZORAL124−64−63
Magnetomorum
Mycobacterium
Nodularia
OLB17
Olleya
Polaromonas
Psychromonas
Roseibacillus
Ruminiclostridium A
Sediminispirochaeta
Steroidobacter
Sulfurimonas
Sulfurovum
Thauera
Treponema E
UBA12014
UBA1847
UBA2284
UBA3006
UBA4055
UBA6092
UBA6803
UBA966
Unclassified

Figure S8

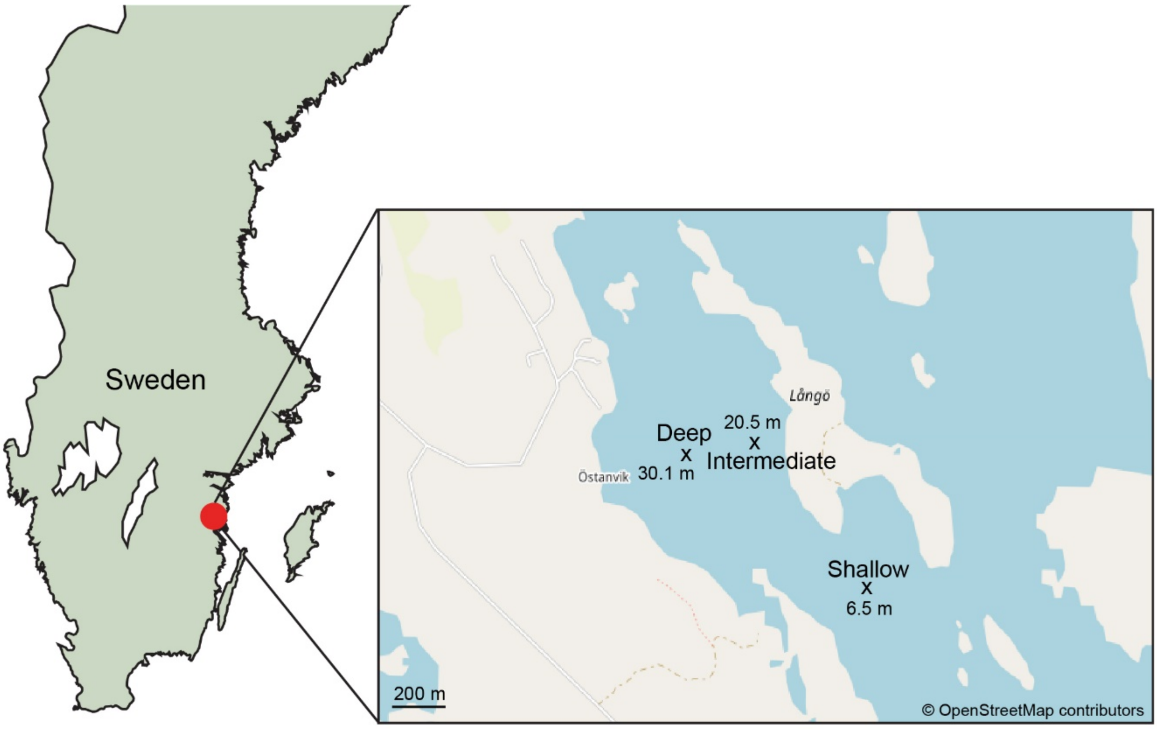

Sweden
Deep
30.1 m
20.5 m
Intermediate
Långö
Östanvik
Shallow
6.5 m
200 m
© OpenStreetMap contributors

Figure S9

Supplemental Table S1

1) Comparison of years within each site with PERMANOVA using Adonis function in vegan package, with Bray-Curtis distances. P-value was corrected with Benjamin-Hochberg correction; 9999 permutations were used, *n=3* per site.

	**F-Model**	**R2**	**p-value**	**BH correction**
shallow	2.09	0.376	0.09	0.188
intermediate	4.33	0.502	0.03*	0.066
deep	3.89	0.474	0.01*	0.033*

2) Comparison of sites within each year (2013 & 2017) with PERMANOVA using Adonis function in the vegan package, with Bray-Curtis distances. 9999 permutations were used, *n*=9.

	F-Model	R2	p-value
2013	2.82	0.484	0.014*
2017	8.58	0.741	0.005**

3) A linear regression model was used to analyze the effects of year and sampling site on bacterial diversity (Shannon´s H index). Within the model, years, sampling sites and replicates have been used as fixed effects, with years and sampling sites as interacting factors and replicates nested within sampling sites. The results of the ANOVA ('anova' function) show if there was a significant effect of the tested factors (a), and pairwise comparisons ('emmeans' function) were used to test for differences between years within each sampling site (b); *n*=18.

(a)

**Diversity Index**	**variable**	**DF**	**F-value**	**p-value**
Shannon H	year	1	7.22	0.036*
	site	2	4.34	0.068
	year:site	2	0.81	0.486
	site:replicate	6	0.94	0.525

(b)

**Diversity Index**	**variable**	**estimate**	**SE**	**df**	**t.ratio**	**p-Value**
Shannon H	2013-2017 shallow	-0.447	0.47	6	-0.950	0.378
	2013-2017 intermediate	-1.217	0.47	6	-2.590	0.041*
	2013-2017 deep	-0.524	0.47	6	-1.116	0.307

***: p<0.001 **: p<0.01 *: p<0.05

4) A linear regression model was used to analyze the effects of year and sampling site on environmental variables. Within the model, years, sampling sites and replicates have been used as fixed effects, with years and sampling sites as interacting factors and replicates nested within sampling sites. The results of the ANOVA ('anova' function) show if there was a significant effect of the tested factors (a), and pairwise comparisons ('emmeans' function) were used to test for differences between years within each sampling site (b); *n*=18.

(a)

Environmental variable	variable	DF	F-value	p-value
nitrate+nitrite	year	1	9.55	0.027*
	site	2	87.12	0.0001***
	year:site	2	12.87	0.010*
	site:replicate	6	2.70	0.147
total iron	year	1	0.05	0.838
	site	2	2.86	0.169
	year:site	2	0.57	0.606
	site:replicate	6	0.79	0.618
sulfate	year	1	2.03	0.213
	site	2	10.09	0.017*
	year:site	2	3.17	0.128
	site:replicate	6	0.92	0.545
phosphate	year	1	0.39	0.557
	site	2	14.15	0.008**
	year:site	2	11.03	0.014*
	site:replicate	6	0.82	0.594
organic matter	year	1	235.21	2.13^{-5}***
	site	2	427.73	2.57^{-6}***
	year:site	2	114.45	6.68^{-5}***
	site:replicate	6	2.80	0.1387

(b)

Environmental variable	variable	estimate	SE	t.ratio	p-value
nitrate+nitrite	2013-2017 shallow	14.53	2.65	5.478	0.002**
	2013-2017 intermediate	-3.42	2.65	-1.293	0.252
	2013-2017 deep	0.50	3.25	0.154	0.883
total iron	2013-2017 shallow	-1.51	2.87	-0.544	0.615
	2013-2017 intermediate	2.59	2.78	0.932	0.404
	2013-2017 deep	1.38	2.27	0.609	0.575
sulfate	2013-2017 shallow	0.38	0.161	2.369	0.064
	2013-2017 intermediate	0.07	0.161	0.490	0.645
	2013-2017 deep	-0.36	0.198	-1.873	0.1256
phosphate	2013-2017 shallow	-5.48	19.1	-0.287	0.785
	2013-2017 intermediate	59.39	19.1	3.115	0.026*
	2013-2017 deep	-68.20	23.3	-2.921	0.033*
organic matter	2013-2017 shallow	-2.133	0.55	-3.819	0.012*
	2013-2017 intermediate	-0.25	0.68	-0.377	0.721
	2013-2017 deep	-11.83	0.55	-21.18	<0.001***

***: p<0.001 **: p<0.01 *: p<0.05

5) Variance inflation factor (VIF) to test for multi-collinearity. The factor shows how well the bacterial communities are explained by the different environmental variables.

Variable	VIF
Oxygen	11.78
Temperature	2.55
Total iron	1.59
Phosphate	2.87
Sulfate	1.47
Nitrate	8.89
OM	3.55

6) ANOVA like permutation test on a RDA to test for significant environmental variables explaining the differences of the bacterial communities. Permutations = 999, n=15, direct model by marginal terms.

Variable	Df	Variance	F-value	p-value
Oxygen	1	3869.9	2.14	0.016*
Temperature	1	3315.6	1.83	0.037*
Total iron	1	876.3	0.48	0.968
Phosphate	1	3044.8	1.68	0.086
Sulfate	1	2683.5	1.48	0.108
OM	1	5406.0	2.99	0.001***

***: p<0.001 **: p<0.01 *: p<0.05

Supplemental Table S3

Rarefied data

Figure 1 | Shannon´s H diversity Index on rarefied data (*n*=18). Data were rarefied based on the smallest sample size *n*=1564 using the rarefy function from the vegan package.

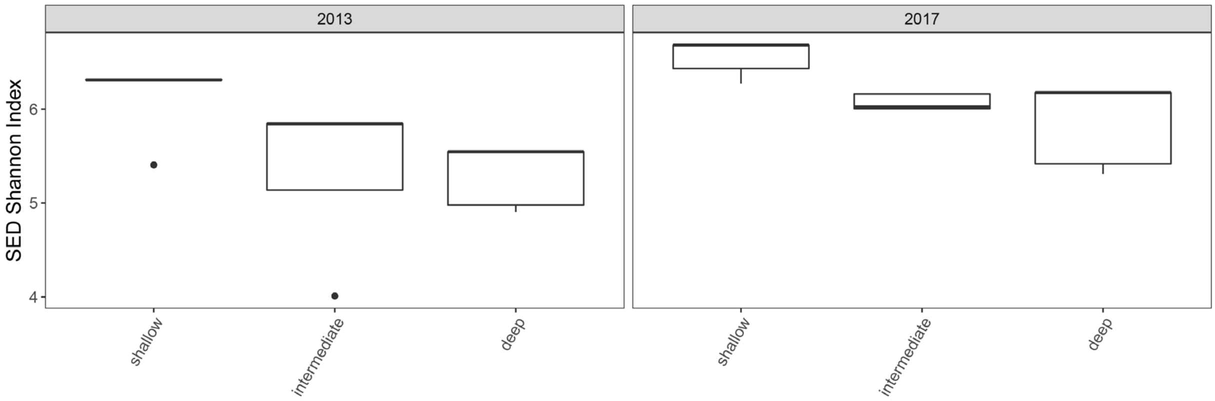

Figure 2 | Non-metric multidimensional scaling (NMDS) based on Bray-Curtis distance (*n*=18). Data were rarefied based on the smallest sample size *n*=1564 using the rarefy function from the vegan package. Stress-value=0.043.

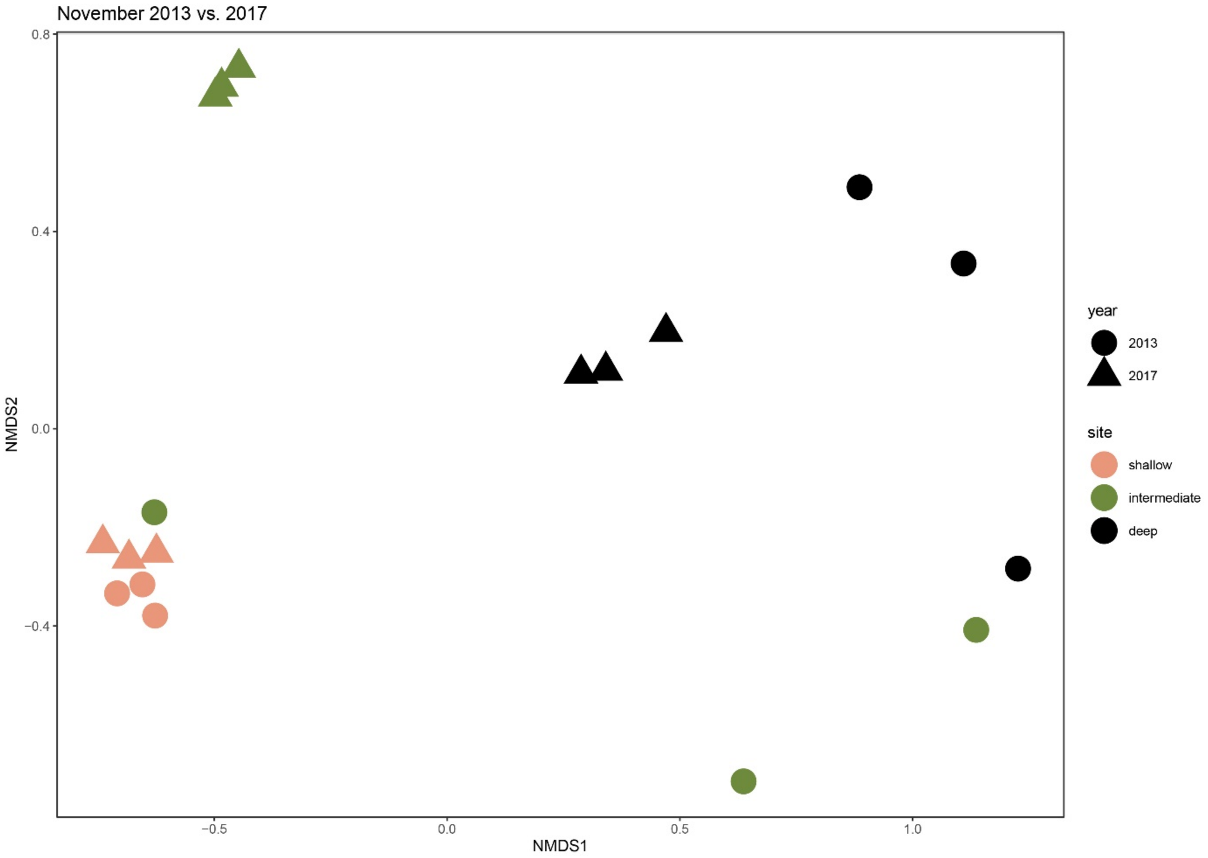

Figure 3 | Bar plot on phylum Level (n=18). Data were rarefied based on the smallest sample size n=1564 using the rarefy function from the vegan package. Each site is shown with all three replicates separated. Taxa with >0.5% relative abundance in 2013 and 2017 are shown; **a** shows bar plot based non-rarefied data; **b** shows bar plots based on rarefied data.

a

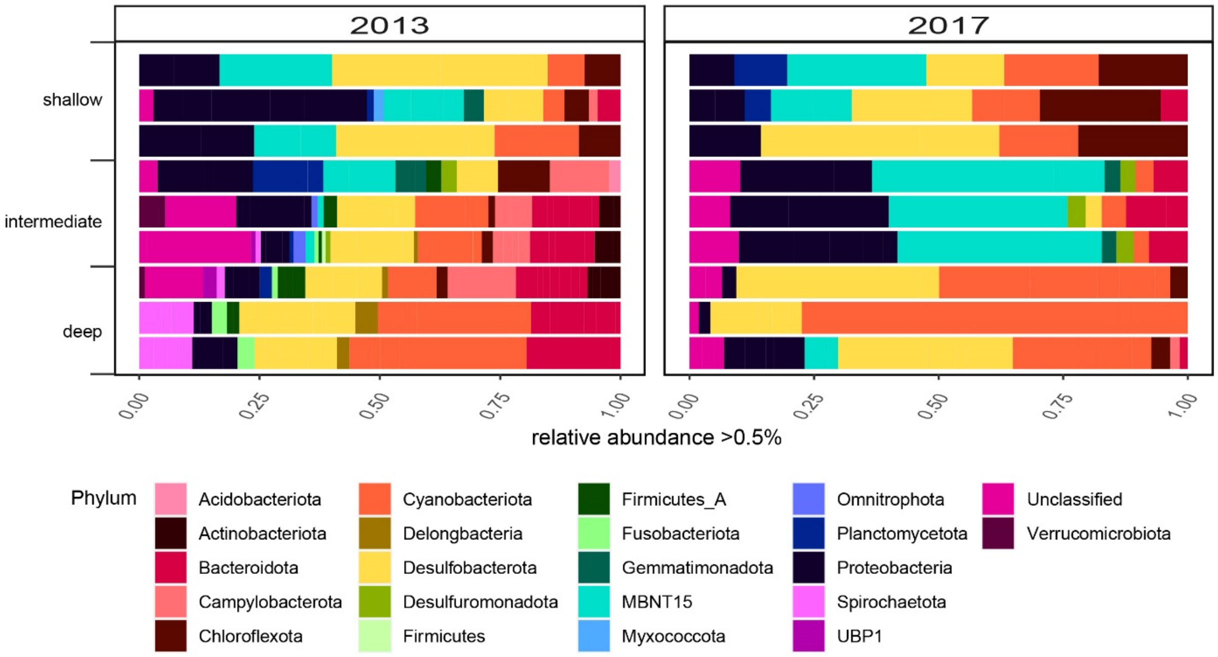

b

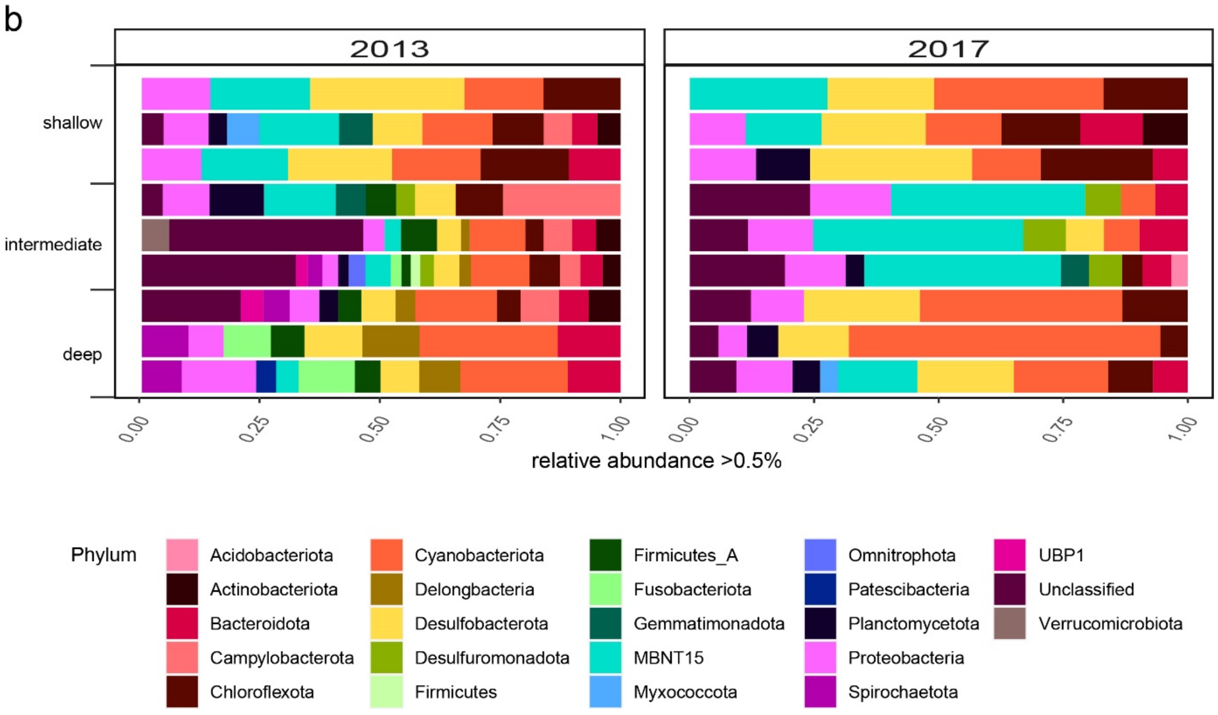

Figure 4 | Bar plot on genus Level (*n*=18). Data were rarefied based on the smallest sample size *n*=1564 using the rarefy function from the vegan package. Each site is shown with all three replicates separated. Taxa with >0.5% relative abundance in 2013 and 2017 are shown; **a** shows bar plot based non-rarefied data; **b** shows bar plots based on rarefied data.

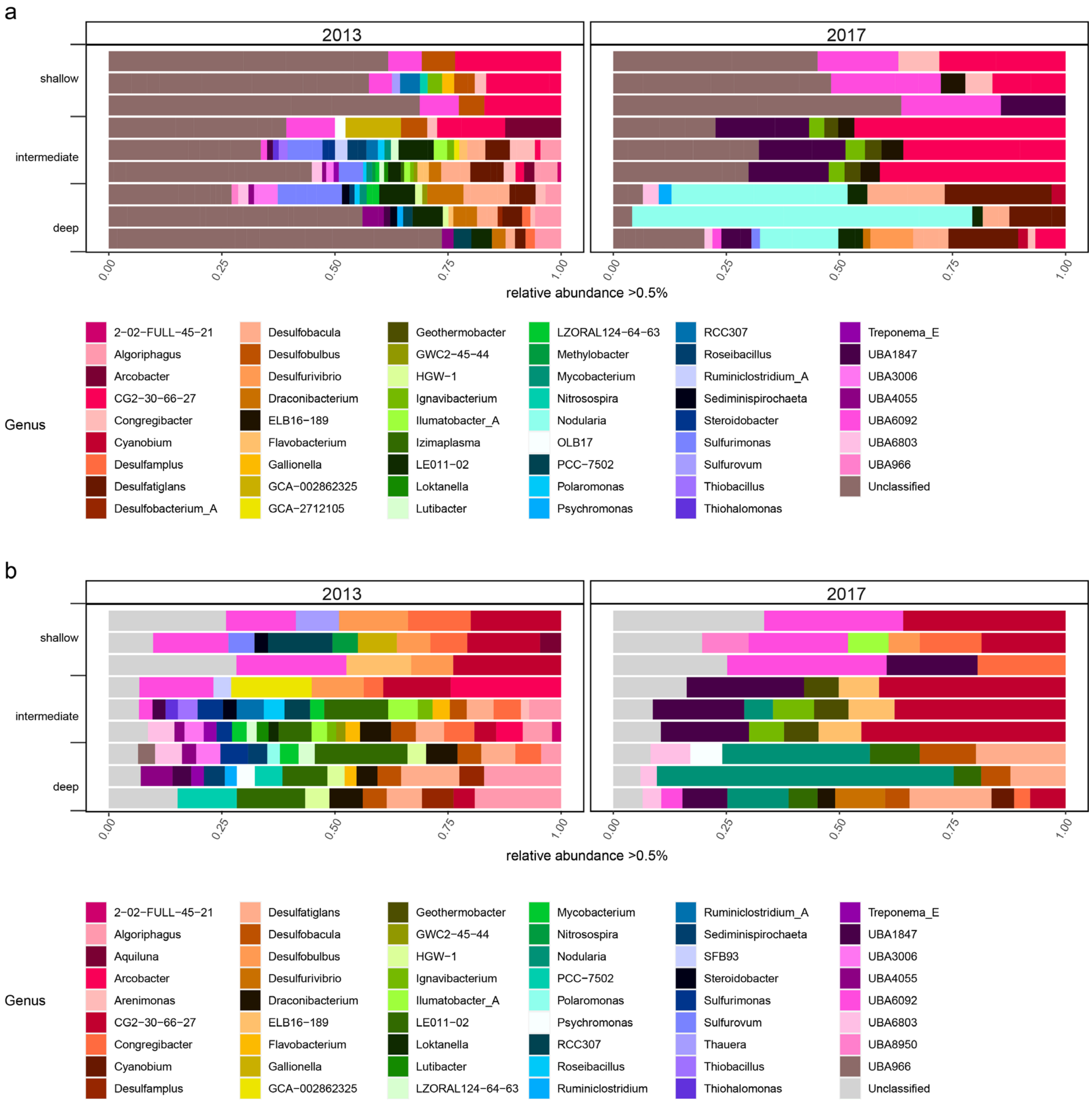

Script Methods S1

#libraries

```
library(tidyverse)
library(vegan)
library(dplyr)
library(phyloseq)
library(gplots)
library(corrplot)
library(adespatial)
library(ggplot2)
library(ggpubr)
library(ggord)
library(ggfortify)
library(GUniFrac)
library(rtk)
library(pheatmap)
library(reshape2)
library(lattice)
library(nlme)
library(DESeq2)
library(apeglm)
library(zinbwave)
library(scran)
library(cowplot)
library(pscl)
library(MASS)
library(data.table)
library(SRS)
library(CoDaSeq)
```

#tables

```
#Meta table for Sediment
meta <- read.csv("meta_SED_TS_Oestanvik.csv", stringsAsFactors = FALSE)

#Counts
countsSED <-  read.table("seqtab_final.txt",stringsAsFactors =FALSE, header=TRUE)%>%
gather(sample,count, 2:ncol(.))%>%filter(count > 0)

#Taxa
taxa <- read.table("tax_final.txt",stringsAsFactors = FALSE, header=TRUE)%>%
	replace_na(list(Kingdom="Unclassified"))%>%
	replace_na(list(Phylum="Unclassified"))%>%
	replace_na(list(Class="Unclassified"))%>%
	replace_na(list(Order="Unclassified"))%>%
	replace_na(list(Family="Unclassified"))%>%
	replace_na(list(Genus="Unclassified"))%>%
	replace_na(list(Species="Unclassified"))%>%
	filter(Family != "Mitochondria") %>%
	filter(Class  != "Chloroplast")
```

```
#####relative abundance and filter everything under 500 counts #####
asvsSED<- countsSED %>%
  # Group by sample, to get access to the group wise sum of counts
  group_by(sample) %>%
  # Filter to keep only rows from samples having at least 500 counts
  filter(sum(count) >= 500) %>%
  # Since we have the data grouped, we can calculate the relative abundance here
  mutate(relab = count/sum(count)) %>%
  ungroup()%>%
    full_join(meta, by = "sample") %>%
  full_join (taxa, by = "sequence")

#Filter for data for November 2013 and November 2017
asvsSED <- asvsSED %>%
        filter(!year %in% c("2014","2015","2016")) %>%
        filter(month=="november")
meta.november <- meta %>%
              filter(year %in% c("2013","2017")) %>%
              filter(month=="november")
```

rarefaction curve, Figure S2

```
#make data in right format (wide)
countsRAW <- asvsSED%>%
  dplyr::select(sequence, sample, count) %>%
  spread(sequence,count, fill= 0) %>%
  remove_rownames() %>%
  column_to_rownames(var = "sample")
#select right meta samples
metanov <- meta %>%
  filter(month=="november")%>%
  filter(year %in% c("2017","2013"))%>%
  arrange(sample)

#chose color for samples
col <- c ("black","darkolivegreen4","darksalmon")
grp <- factor(metanov$site, levels= c("deep","intermediate","shallow"))
cols <- col[grp]

#smallest sample
(raremax <- min(rowSums(countsRAW)))

##Figure
out <-  rarecurve(countsRAW, step = 20, sample = raremax, col = cols , cex = 0.6, label=TRUE)

#Rarefy data to check data viability

####rarefy community data
raremin <- min(rowSums(countsRAW))

M = as.matrix(t(countsRAW))
densityOfSample1 = density(M[,1])
hist(M[,1], nclass = 50)
```

```
S_densities = apply(M,2,density)

##Rarefried table
M2 = t(rrarefy(t(M),sample=raremin))

##Check sum
colSums(M2)##All same sum

##Either use the rarefied data or the count data
```

#**Alpha diversity, Figure S2**

```
##raw counts from  SED
example_input_data <- asvsSED %>%  dplyr::select(sequence, sample, count)%>%  #select ISV,
sample and relab
  spread(sample,count, fill= 0) %>% #wide format with sequence and relab, filling gaps with 0
  remove_rownames() %>% #remove of row names
  column_to_rownames(var = "sequence")

#(e.g. species counts of the library with the lowest sequencing depth):
Cmin <- min(colSums(example_input_data))
Cmin

SRS_output <- SRS(data = example_input_data, Cmin = Cmin)
SRS_output

###Shannon Diversity

SRSshannon<- SRS_output %>%
  t()%>%
  data.frame()%>%
  rownames_to_column(var="sample")%>%
  plyr::ddply(~sample, function(x) {vegan::diversity(x[-1], index="shannon")}) %>%
  dplyr::rename(shannon= V1) %>% #Shannon
  left_join(asvsSED, by="sample")

SRSshannon$site<-factor(SRSshannon$site, levels = c("shallow","intermediate","deep"))


shan <- SRSshannon %>%
  ggplot(aes(x = site, y = shannon)) +
  geom_boxplot() +
  labs(x = "", y = "SED Shannon Index") +
  theme_bw() +
  facet_wrap(~year, nrow=1)+
  theme(text = element_text(size = 14)) +
  theme(panel.grid = element_blank(), axis.title.x = element_text(size = 12))+
   theme(
    axis.text.x = element_text(angle = 60, hjust = 1)
  )
Shan
```

Diversity statistics

```
B10 <- lm(shannon ~   year * site +  site/replicate , data=SRSshannon,  method="REML")

##running test with emmeans
library(emmeans)

emmeans(B10, spec="year",by="site", contr="pairwise")

plot(B10)
E <- resid(B10)
hist(E)
```

#Environmental variables, Figure 1

```
##environemtal data
chem.meta <- read.csv("chemistry_november_2013_2017.csv", stringsAsFactors = FALSE)

##Making a plot for each variable
###Phosphate

chem.meta$site <- factor(chem.meta$site, levels = c("shallow","intermediate","deep"))

phos <- chem.meta %>%
        ggplot(aes(x=factor(year), y=mean.phos, fill=site))+
         geom_bar(stat="identity",position=position_dodge(width=0.9))+
           scale_fill_manual(values=c("darksalmon","darkolivegreen4","black"))+
         geom_errorbar(aes(ymin=mean.phos-err.phos, ymax=mean.phos+err.phos, group=site),
width=0.25, color="grey", position=position_dodge(width = 0.9) ) +
         facet_wrap(~site)+
         ggtitle("Phosphate on Porewater")+
         xlab("year")+
          ylab("µM")+
           theme(
   axis.text.x = element_text(angle = 25, hjust = 1)
  )+
   theme(panel.grid.major = element_blank(), panel.grid.minor = element_blank(),
panel.background = element_blank(), axis.line = element_line(colour = "black"))

###pH
 pH<-     chem.meta %>%
         ggplot(aes(x=factor(year), y=mean.ph, fill=site))+
         geom_bar(stat="identity",position=position_dodge(width=0.9))+
           scale_fill_manual(values=c("darksalmon","darkolivegreen4","black"))+
         geom_errorbar(aes(ymin=mean.ph-err.ph, ymax=mean.ph+err.ph, group=site), width=0.25,
color="grey", position=position_dodge(width = 0.9) ) +
         facet_wrap(~site)+
         ggtitle("pH on Porewater")+
         xlab("year")+
          ylab("pH")+
           theme(
   axis.text.x = element_text(angle = 25, hjust = 1)
```

```
  )+
   theme(panel.grid.major = element_blank(), panel.grid.minor = element_blank(),
panel.background = element_blank(), axis.line = element_line(colour = "black"))


###total iron

    totIron <-    chem.meta %>%
                ggplot(aes(x=factor(year), y=mean.iron, fill=site))+
                geom_bar(stat="identity",position=position_dodge(width=0.9))+
                  scale_fill_manual(values=c("darksalmon","darkolivegreen4","black"))+
                geom_errorbar(aes(ymin=mean.iron-err.iron, ymax=mean.iron+err.iron, group=site),
width=0.25, color="grey", position=position_dodge(width = 0.9) ) +
                facet_wrap(~site)+
                ggtitle("Total Iron on Porewater")+
                xlab("year")+
                 ylab("µM")+
                  theme(
    axis.text.x = element_text(angle = 25, hjust = 1)
  )+
   theme(panel.grid.major = element_blank(), panel.grid.minor = element_blank(),
panel.background = element_blank(), axis.line = element_line(colour = "black"))


###sulfate
     sulf <-  chem.meta %>%
                ggplot(aes(x=factor(year), y=mean.sulf, fill=site))+
                geom_bar(stat="identity",position=position_dodge(width=0.9))+
                  scale_fill_manual(values=c("darksalmon","darkolivegreen4","black"))+
                geom_errorbar(aes(ymin=mean.sulf-err.sulf, ymax=mean.sulf+err.sulf, group=site),
width=0.25, color="grey", position=position_dodge(width = 0.9) ) +
                facet_wrap(~site)+
                ggtitle("Sulfate on Porewater")+
                xlab("year")+
                 ylab("mM")+
                  theme(
    axis.text.x = element_text(angle = 25, hjust = 1)
  )+
   theme(panel.grid.major = element_blank(), panel.grid.minor = element_blank(),
panel.background = element_blank(), axis.line = element_line(colour = "black"))

###nitrate


nit <-   chem.meta %>%
                ggplot(aes(x=factor(year), y=mean.nit, fill=site))+
                geom_bar(stat="identity",position=position_dodge(width=0.9))+
                  scale_fill_manual(values=c("darksalmon","darkolivegreen4","black"))+
                geom_errorbar(aes(ymin=mean.nit-err.nit, ymax=mean.nit+err.nit, group=site),
width=0.25, color="grey", position=position_dodge(width = 0.9) ) +
                facet_wrap(~site)+
                ggtitle("Nitrate/Nitrite on Porewater")+
                xlab("year")+
```

```
                 ylab("µM")+
                  theme(
   axis.text.x = element_text(angle = 25, hjust = 1)
  )+
   theme(panel.grid.major = element_blank(), panel.grid.minor = element_blank(),
panel.background = element_blank(), axis.line = element_line(colour = "black"))

####OM
om <-     chem.meta %>%
        ggplot(aes(x=factor(year), y=mean.om, fill=site))+
                 geom_bar(stat="identity",position=position_dodge(width=0.9))+
                   scale_fill_manual(values=c("darksalmon","darkolivegreen4","black"))+
                 geom_errorbar(aes(ymin=mean.om-err.om, ymax=mean.om+err.om, group=site),
width=0.25, color="grey", position=position_dodge(width = 0.9) ) +
                 facet_wrap(~site)+
                 ggtitle("organic matter on Porewater")+
                 xlab("year")+
                  ylab("%")+
                   theme(
   axis.text.x = element_text(angle = 25, hjust = 1)
  )+
   theme(panel.grid.major = element_blank(), panel.grid.minor = element_blank(),
panel.background = element_blank(), axis.line = element_line(colour = "black"))


###oxygen
oxy <-  chem.meta %>%
                 ggplot(aes(x=factor(year), y=oxygen1, fill=site))+
                 geom_bar(stat="identity",position=position_dodge(width=0.9))+
                   scale_fill_manual(values=c("darksalmon","darkolivegreen4","black"))+
                 facet_wrap(~site)+
                 ggtitle("Oxygen")+
                 xlab("year")+
                  ylab("mg/L")+
                   theme(
   axis.text.x = element_text(angle = 25, hjust = 1)
  )+
   theme(panel.grid.major = element_blank(), panel.grid.minor = element_blank(),
panel.background = element_blank(), axis.line = element_line(colour = "black"))

###Temperature
temp <-  chem.meta %>%
                 ggplot(aes(x=factor(year), y=temperature, fill=site))+
                 geom_bar(stat="identity",position=position_dodge(width=0.9))+
                   scale_fill_manual(values=c("darksalmon","darkolivegreen4","black"))+
                 facet_wrap(~site)+
                 ggtitle("temperature")+
                 xlab("year")+
                  ylab("°C")+
                   theme(
   axis.text.x = element_text(angle = 25, hjust = 1)
  )+
   theme(panel.grid.major = element_blank(), panel.grid.minor = element_blank(),
```

```
panel.background = element_blank(), axis.line = element_line(colour = "black"))

###Salinity
sal <-  chem.meta %>%
                ggplot(aes(x=factor(year), y=salinity, fill=site))+
                geom_bar(stat="identity",position=position_dodge(width=0.9))+
                  scale_fill_manual(values=c("darksalmon","darkolivegreen4","black"))+
                facet_wrap(~site)+
                ggtitle("salinity")+
                xlab("year")+
                 ylab(".%")+
                  theme(
    axis.text.x = element_text(angle = 25, hjust = 1)
  )+
   theme(panel.grid.major = element_blank(), panel.grid.minor = element_blank(),
panel.background = element_blank(), axis.line = element_line(colour = "black"))


ggarrange(phos,  totIron, sulf, nit, om, ncol=2,nrow=3)

ggarrange(pH,oxy,temp,sal, ncol=2,nrow=2)


##Statistical significance test

B10 <- lm(organicmatter ~   year * site +  site/replicate , data=meta.november,  method="REML")
anova(B10)
emmeans(B10, spec="year",by="site", contr="pairwise")

plot(B10)

E <- resid(B10)
hist(E)
```

#NMDS, Figure 2

```
asvs_subsed <-  asvsSED %>%
  filter(month=="november")%>%
  filter(year %in%c("2013","2017"))%>%
  dplyr::select(sequence,sample,relab) %>% #select ISV, sample and relab
  spread(sequence,relab, fill= 0) %>% #wide format with sequence and relab, filling gaps with 0
  remove_rownames() %>% #remove of row names
  column_to_rownames(var = "sample")


#NMDS
 # calculate bray curtis distances with metaMDS function, double check autotransform and trace in metaMDS help!!!
nmds.asvs.h.bc <- metaMDS(asvs_subsed ,
                          distance = "bray",
                          k = 3,
                          trymax = 50,
                          autotransform = FALSE, # set to false when using normalized data from relab
```

```
                        trace = FALSE)


nmds.seed.asvs.bc.df <- as.data.frame(nmds.asvs.h.bc$points) %>%
      rownames_to_column(var ="sample") %>%
       dplyr::rename(NMDS1 = MDS1, NMDS2 = MDS2)

     # check the stress plot should be a nearly linear fit
nmds.asvs.h.bc.stress <- stressplot(nmds.asvs.h.bc)


p <- nmds.seed.asvs.bc.df %>% inner_join (meta.november, by="sample")%>%
  ungroup()%>%
  ggplot( aes(x=NMDS1, y=NMDS2, color=site, shape=factor(year), label=sample)) +
  geom_point(size = 8) +
 # geom_label(aes(group=sample))+
  # stat_ellipse(aes(color=oxygenstate))+
  scale_color_manual(values=c( "darksalmon","darkolivegreen4","black"))+
  theme_bw() +
  labs(title = "november 2013 vs. 2017")

p
```

#**Bray Curtis dissimilarity, Figure S3**

```
asvs_subsed.nov <-  asvsSED %>%
  filter(month=="november")%>%
  dplyr::select(sequence,sample,relab) %>% #select ISV, sample and relab
  spread(sequence,relab, fill= 0) %>% #wide format with sequence and relab, filling gaps with 0
  remove_rownames() %>% #remove of row names
  column_to_rownames(var = "sample")


braydist <- vegdist(asvs_subsed.nov, method = "bray", upper=TRUE)
bray.matrix <- as.matrix(braydist)
melted_bray <- melt(bray.matrix)

####HEATmap with GGPLOT

# Get lower triangle of the correlation matrix
  get_lower_tri<-function(bray.matrix){
    bray.matrix[upper.tri(bray.matrix)] <- NA
    return(bray.matrix)
  }
  # Get upper triangle of the correlation matrix
  get_upper_tri <- function(bray.matrix){
    bray.matrix[lower.tri(bray.matrix)]<- NA
    return(bray.matrix)
  }

  upper_tri <- get_upper_tri(bray.matrix)

  # Melt the correlation matrix
```

```
melted_cormat <- melt(upper_tri, na.rm = TRUE)

# Heatmap
ggheatmap <-ggplot(data = melted_cormat, aes(Var2, Var1, fill = value))+
 geom_tile(color = "white")+
 scale_fill_gradient2(low = "yellow", high = "darkorchid4", mid = "green",
   midpoint = 0.5, limit = c(0,1), space = "Lab",
   name="Bray Curtis Dissimilarity") +
  theme_minimal()+
 theme(axis.text.x = element_text(angle = 45, vjust = 1,
    size = 12, hjust = 1))+
 coord_fixed()+
  geom_text(aes(Var2, Var1, label =sprintf("%0.2f",round(value, digits=2))), color = "black", size =
4)+
  theme(
  axis.title.x = element_blank(),
  axis.title.y = element_blank(),
  panel.grid.major = element_blank(),
  panel.border = element_blank(),
  panel.background = element_blank(),
  axis.ticks = element_blank(),
  legend.justification = c(1, 0),
  legend.position = c(0.6, 0.7),
  legend.direction = "horizontal")+
  guides(fill = guide_colorbar(barwidth = 7, barheight = 1,
              title.position = "top", title.hjust = 0.5))

ggheatmap
```

#RDA, Figure S5

```
#Transform the data (centered-log transformation)
asvsclr_SED <-asvs_subsed %>%
  t() %>%
  data.frame() %>%
  cmultRepl(method = 'CZM', delta = 0.5, output = 'p-counts') %>%
  codaSeq.clr(samples.by.row = FALSE) %>%
  data.frame() %>%
  tibble::rownames_to_column('sequence') %>%
  gather(sample, clr, 2:ncol(.)) %>%
  left_join(meta.november, by = c('sample')) %>%
  replace_na(list(count = 0))

#Make the table into the right format
mat <- asvsclr_SED %>%
  dplyr::select(sequence, sample, clr)%>%
  spread(sequence, clr, fill=0)%>%
  remove_rownames()%>%
  column_to_rownames(var="sample")

#Prepare the meta table
mat_meta <- meta.november %>%
    filter(!sample %in% c("E3","E6","E8"))%>%
```

```
  dplyr::select(sample, oxygen1, temperature,Irontot, Phosphate, Sulphate, Nitrate, organicmatter
)%>%
  remove_rownames()%>%
  column_to_rownames(var="sample")

#Run the RDA, with selected env.var.
rda_tree = rda(mat ~ oxygen1 + temperature+ Irontot+ Phosphate+Sulphate+ Nitrate+ organicmatter,
data=mat_meta)

rda_tree

#ANOVA like Permutation test
set.seed(10)
anova(rda_tree, by="terms", permutations = 999)

#Calculate the % of each CAP
dpRDA.eigs    <- rda_tree$CCA$eig %>%
  data.frame() %>%
  tibble::rownames_to_column('rda_tree') %>%
  #rename(eigval = ".") %>%
  mutate(propexpl = ./sum(.))
dpRDA.eigs.uncon    <- rda_tree$CA$eig %>%
  data.frame() %>%
  tibble::rownames_to_column('rda_tree') %>%
 # rename(eigval = 2) %>%
  mutate(propexpl = ./sum(.))

expl_var <- c(dpRDA.eigs , dpRDA.eigs.uncon)


#PLOT
#Extract site data first
scrs <- scores(rda_tree, display=c("sp","wa","lc","bp","cn"))
df_sites<-data.frame(scrs$sites,t(as.data.frame(strsplit(rownames(scrs$sites),"_"))))
colnames(df_sites)<-c("CAP1","CAP2","sample")

df_sites <- df_sites %>%
inner_join(meta.november, by="sample")

#Draw sites
p<-ggplot()
p<-p+geom_point(data=df_sites,aes(CAP1,CAP2,colour=site,shape=factor(year), size=5))+
  scale_color_manual(values=c(  "black","darkolivegreen4","darksalmon"))

#Draw biplots
multiplier <- vegan:::ordiArrowMul(scrs$biplot*0.2)

df_arrows<- scrs$biplot*multiplier
colnames(df_arrows)<-c("CAP1","CAP2")
df_arrows=as.data.frame(df_arrows)

p<-p+geom_segment(data=df_arrows, aes(x = 0, y = 0, xend = CAP1, yend = CAP2),
                 arrow = arrow(length = unit(0.2, "cm")))
```

```
p<-p+geom_text(data=as.data.frame(df_arrows*1.2),aes(CAP1, CAP2, label =
rownames(df_arrows)))+
  theme_classic()
p
```

#perMANOVA, significance testing between years, Table S1

```
###PERMANOVA TEST WITH ADONIS2 Compare years for each site, including replicates, and
Benjamin Hochberg correction

asvs_subsed.nov <-  asvsSED %>%
 # filter(year=="2017")%>%
 filter(month=="november")%>%
 filter(site=="shallow")%>%
  dplyr::select(sequence,sample,relab) %>% #select ISV, sample and relab
  spread(sequence,relab, fill= 0) %>% #wide format with sequence and relab, filling gaps with 0
  remove_rownames() %>% #remove of row names
  column_to_rownames(var = "sample")

meta.november_ad <- meta.november %>%
                filter(site=="shallow")

braydist <- vegdist(asvs_subsed.nov, method = "bray", upper=TRUE)
bray.matrix <- as.matrix(braydist)

bray.sig <- adonis2(bray.matrix ~year+replicate, data= meta.november_ad, method="bray",
permutations = 9999)

x <- bray.sig$"Pr(>F)"

p.adjust(as.matrix(x), method = "BH")
```

#Differential abundance analysis with Deseq2

```
#Using Zero-Inflated Negative Binominal Model to reduce excess of Zeros in Dataset before using
Deseq for Differential ABundance analysis
###Make an phyloseq object
OTU <- asvsSED%>%
  group_by(sequence, sample)%>%
  summarise(count=sum(count))%>%
  dplyr::select(sequence, sample, count)%>%
  spread(sample, count,fill=0 ) %>%
  column_to_rownames("sequence")

TAXA <- taxa %>% semi_join(asvsSED,
by=c("sequence","Kingdom","Family","Order","Class","Genus","Species"))%>%
  remove_rownames()%>%
  column_to_rownames(var="sequence")

SAMPLES <- meta.november %>%
  remove_rownames() %>%
  column_to_rownames(var="sample")
```

```
OTU <- as.matrix(OTU)
TAXA <- as.matrix(TAXA)

  OTU = otu_table(OTU, taxa_are_rows = TRUE)
  TAX = tax_table(TAXA)
  samples = sample_data(SAMPLES)

  carbom <- phyloseq(OTU, TAX, samples)
  carbom

###Get rid of low abundant Taxa
species_counts_df <- data.frame(otu_table(carbom))
(sum(colSums(species_counts_df == 0))) / (nrow(species_counts_df) * ncol(species_counts_df))

###~ 85 % zeros in our matrix, now we are droping taxa not seen in at least 20 % of the samples
(carbom <- filter_taxa(carbom, function(x) sum(x > 0) > (0.2*length(x)), TRUE))
##From 9099 Taxa there are only 1844 Taxa left, means 1844 species or ASVs are seen in 20% of the
samples.


###Assessing zero proportion after filtering
species_counts_df <- data.frame(otu_table(carbom))
species_counts_df <- data.frame(t(species_counts_df))

###Proportion of Zeros for each ASV
species_counts_df %>%
  mutate_all(function(x) ifelse(x == 0, 1, 0)) %>%
  summarise_all(function(x) mean(x)) %>%
  t(.) %>%
  data.frame(.) %>%
  dplyr::rename("prop_zeros" = ".") %>%
  ggplot(data = ., aes(x = prop_zeros)) + geom_histogram(bins = 50) +
  scale_x_reverse() +
  labs(x = "\nProportion of Zeros for Each Species", y = "Count\n")


###read count distirbution
set.seed(123)
species_counts_df %>%
  t(.) %>%
  data.frame(.) %>%
  rownames_to_column(var = "Species") %>%
  mutate(rnum = rnorm(n = nrow(.))) %>%
  dplyr::arrange(rnum) %>%
  dplyr::slice(1:12) %>%
  column_to_rownames(var = "Species") %>%
  dplyr::select(-rnum) %>%
  t(.) %>%
  data.frame(.) %>%
  pivot_longer(cols = everything(), names_to = "Species", values_to = "Counts") %>%
  dplyr::arrange(Species) %>%
  ggplot(data = ., aes(x = Counts)) + geom_histogram(bins = 100) +
```

```
  labs(x = "\nRead Count Distribution", y = "Count\n") +
  facet_wrap(~ Species, scales = "fixed")


dds_zinbwave <- phyloseq_to_deseq2(carbom, ~ year+site/replicate)

ds_zinbwave <- zinbwave(dds_zinbwave,
                X="~ 1",
                epsilon = 1e10,
                verbose = TRUE,
                K = 0,
                observationalWeights = TRUE,
                BPPARAM = BiocParallel::SerialParam())


dds_zinb <- DESeqDataSet(dds_zinbwave, design = ~ year+site/replicate)
   dds_zinb$group <- factor(paste0(dds_zinb$site, dds_zinb$year))
   design(dds_zinb)<- ~group
dds_zinb <- estimateSizeFactors(dds_zinb, type="poscounts")
scr <- computeSumFactors(dds_zinb)

sizeFactors(dds_zinb) <- sizeFactors(scr)


###Fit the model use the LRT
dds_zinb <- DESeq(dds_zinb, test="LRT", reduced=~1, sfType="poscounts",
               minmu=1e-6, minReplicatesForReplace=Inf, fitType = "local")

plotMA(dds_zinb)
plotDispEsts(dds_zinb)

###Make contrast so you can differentiate between the groups you are looking for
   resultsNames(dds_zinb)
   deepDE2 <- results(dds_zinb, contrast=c("group","deep2013","deep2017"))
   shallDE2 <-results(dds_zinb, contrast=c("group","shallow2013","shallow2017"))
   interDE2 <-results(dds_zinb, contrast=c("group","intermediate2013","intermediate2017"))

##make it readable

  #  shallDE2_results <- data.frame(deepDE2)


  # baseMeanPerLvl <- sapply( levels(dds$group), function(lvl)
rowMeans(counts(dds,normalized=TRUE)[,dds$group == lvl] ) )
   #shallDE2_results <-merge(shallDE2_results,baseMeanPerLvl, by="row.names")
   #shallDE2_results<- shallDE2_results[,c(1,2,8,9,3,4,5,6,7)]

 # sorted_deepDE2_results <- shallDE2_results[order(-shallDE2_results$baseMean),]
  # colnames(sorted_deepDE2_results)[1] <- "sequence"

  #sorted_taxa <- sorted_deepDE2_results %>% left_join(taxa, by="sequence")
```

```
 # write.csv(sorted_taxa ,
"Deseq_differential_abundance_shallow_ASV_level_zero_inflated_count_model_filtered_low_abund
ance.csv")
```

#Differential abundance, Figure 3 & 4

```
##Genus Level
##deep
deepfidd <-
read.csv("Deseq_differential_abundance_deep_ASV_level_zero_inflated_count_model_filtered_low_
abundance.csv", stringsAsFactors = FALSE)

deepfilt <- deepfidd %>% filter(padj <0.05) %>% #filter(!between(log2FoldChange, -5, 5))%>%
        dplyr::select(-deep2013,-deep2017)
deeprest <- deepfidd %>%anti_join(deepfilt, by="sequence") %>% mutate(Genus =
"xx_not_significant")%>%
        dplyr::select(-deep2013,-deep2017)


abunddeep <- rbind(deepfilt,deeprest)

asvdeep <- asvsSED %>% filter(site%in% "deep")

deepfull <- abunddeep %>%
      left_join(asvdeep,
by=c("sequence","Kingdom","Phylum","Order","Class","Family","Species"))%>%
      filter(site%in%"deep")


###intermediate

intermediatefidd <-
read.csv("Deseq_differential_abundance_intermediate_ASV_level_zero_inflated_count_model_filtere
d_low_abundance.csv", stringsAsFactors = FALSE)

interfilt <- intermediatefidd %>% filter(padj <0.05) %>%  dplyr::select(-X)%>%
        dplyr::select(-intermediate2013,-intermediate2017)


interrest <- intermediatefidd %>%anti_join(interfilt, by="sequence") %>% mutate(Genus =
"xx_not_significant")%>% dplyr::select(-X)%>%
        dplyr::select(-intermediate2013,-intermediate2017)

abundinter <- rbind(interfilt,interrest)

asvinter <- asvsSED %>% filter(site%in% "intermediate")

interfull <- abundinter %>%
        left_join(asvinter,
by=c("sequence","Kingdom","Phylum","Order","Class","Family","Species"))%>%
        filter(site%in%"intermediate")
###shallow
```

```
shallfidd <-
read.csv("Deseq_differential_abundance_shallow_ASV_level_zero_inflated_count_model_filtered_lo
w_abundance.csv", stringsAsFactors = FALSE)

shallfilt <- shallfidd %>% filter(padj <0.05) %>%
        dplyr::select(-shallow2013,-shallow2017)

shallrest <- shallfidd %>%anti_join(shallfilt, by="sequence") %>% mutate(Genus =
"xx_not_significant")%>%
        dplyr::select(-shallow2013,-shallow2017)

abundshall <- rbind(shallfilt,shallrest)

asvshall <- asvsSED %>% filter(site%in% "shallow")

shallfull <- abundshall %>%
        left_join(asvshall,
by=c("sequence","Kingdom","Phylum","Order","Class","Family","Species"))%>%
        filter(site%in%"shallow")


###combined all

abund.all <- rbind(shallfull, interfull, deepfull)


###rest not significant
shall.not.sig <- shallrest%>%
        left_join(asvshall,
by=c("sequence","Kingdom","Phylum","Order","Class","Family","Species"))%>%
        filter(site%in%"shallow")
inter.not.sig <- interrest%>%
        left_join(asvinter,
by=c("sequence","Kingdom","Phylum","Order","Class","Family","Species"))%>%
        filter(site%in%"intermediate")
deep.not.sig <- deeprest%>%
        left_join(asvdeep,
by=c("sequence","Kingdom","Phylum","Order","Class","Family","Species"))%>%
        filter(site%in%"deep")


abund.all.rest <- rbind(shall.not.sig, inter.not.sig, deep.not.sig)

###getting 0.5 % and others

abund.all0.5 <- abund.all%>% filter(relab >0.005)
abund.allrest <- abund.all %>%anti_join(abund.all0.5, by="sequence") %>% mutate(Genus.x =
"xxx_Others")

###combined with others under 0.5 percent and significant over 0.5 percent

abund.all.combined <- rbind(abund.all0.5, abund.allrest)
```

```
###Want to get all non significant in one group and then others significant <0.5 % together

###signifcant within others group
sig.others <- abund.allrest %>% anti_join(abund.all.rest, by="sequence")
##not significant ones within others group
non.sig.others <- abund.all.rest %>% anti_join(sig.others, by="sequence")%>%mutate(Genus.x
="xx_not_significant")

###combine both tables above with the tables over 0.5%

all.combined.final <- rbind(abund.all0.5,sig.others,non.sig.others)##final table


##using Genus X

abund.all$site<-factor(abund.all$site, levels = c("shallow","intermediate","deep"))

all.combined.final  %>%
ggplot(aes(x = sample, y = relab, fill =Genus.x)) +
geom_bar( position="fill", stat="identity") +
coord_flip() +

scale_fill_manual(values=c("#f8004b","#00fad5","#5d00b6","#93ff6c","#c751ff","#77c600","#427cff
","#fff86b","#000f5b","#ff9b1d","#00a7fd","#cf000f","#01e7e3","#d85100",
"#40ddff","#7c000d","#01b981","#8f007d","#007c0e","#ff8af0","#005808","#d59cff","#b79a00","#0
03670","#ffe98b","#8f0060","#deffaa","#8d003c","#b6ffd5","#470019","#d7fff9",
"#080003","#ffeec3","#002945","#ffd190","#1d1600","#eef0ff","#571900","#a5ccff","#843300","#dc
ccff","#2a3500","#ff9cdf","#004d33","#ff76b1","#018887","#ff9580","#00718e","#907000"))+
theme(
   legend.position = 'bottom'
  )+
  xlab('bay') + ylab('relative abundance >0.5%') +
  theme(
   axis.text.x = element_text(angle = 60, hjust = 1)
  )+
  facet_wrap(~year)+
  theme(legend.position="bottom")



only.sig <- all.combined.final %>% filter(!Genus.x %in% c("xx_not_significant","xxx_Others"))

only.sig  %>%
ggplot(aes(x = sample, y = relab, fill =Genus.x)) +
geom_bar( position="fill", stat="identity") +
coord_flip() +

scale_fill_manual(values=c("#f8004b","#00fad5","#5d00b6","#93ff6c","#c751ff","#77c600","#427cff
","#fff86b","#000f5b","#ff9b1d","#00a7fd","#cf000f","#01e7e3","#d85100",
"#40ddff","#7c000d","#01b981","#8f007d","#007c0e","#ff8af0","#005808","#d59cff","#b79a00","#0
03670","#ffe98b","#8f0060","#deffaa","#8d003c","#b6ffd5","#470019","#d7fff9",
```

```
"#080003","#ffeec3","#002945","#ffd190","#1d1600","#eef0ff","#571900","#a5ccff","#843300","#dc
ccff","#2a3500","#ff9cdf","#004d33","#ff76b1","#018887","#ff9580","#00718e",
"#907000"))+
theme(
   legend.position = 'bottom'
  )+
  xlab('bay') + ylab('relative abundance >0.5%') +
  theme(
   axis.text.x = element_text(angle = 60, hjust = 1)
  )+
  facet_wrap(~year)+
  theme(legend.position="bottom")


###Phylum Level

###deep
deepfidd <-
read.csv("Deseq_differential_abundance_deep_ASV_level_zero_inflated_count_model_filtered_low_
abundance.csv", stringsAsFactors = FALSE)

deepfilt <- deepfidd %>% filter(padj <0.05) %>%
            dplyr::select(-deep2013,-deep2017)
deeprest <- deepfidd %>%anti_join(deepfilt, by="sequence") %>% mutate(Phylum =
"xx_not_significant")%>%
            dplyr::select(-deep2013,-deep2017)


abunddeep <- rbind(deepfilt,deeprest)

asvdeep <- asvsSED %>% filter(site%in% "deep")

deepfull <- abunddeep %>%
           left_join(asvdeep,
by=c("sequence","Kingdom","Genus","Order","Class","Family","Species"))%>%
           filter(site%in%"deep")


###intermediate

intermediatefidd <-
read.csv("Deseq_differential_abundance_intermediate_ASV_level_zero_inflated_count_model_filtere
d_low_abundance.csv", stringsAsFactors = FALSE)

interfilt <- intermediatefidd %>% filter(padj <0.05) %>%  dplyr::select(-X)%>%
            dplyr::select(-intermediate2013,-intermediate2017)


interrest <- intermediatefidd %>%anti_join(interfilt, by="sequence") %>% mutate(Phylum =
"xx_not_significant")%>% dplyr::select(-X)%>%
            dplyr::select(-intermediate2013,-intermediate2017)

abundinter <- rbind(interfilt,interrest)
```

```
asvinter <- asvsSED %>% filter(site%in% "intermediate")

interfull <- abundinter %>%
            left_join(asvinter,
by=c("sequence","Kingdom","Genus","Order","Class","Family","Species"))%>%
             filter(site%in%"intermediate")
###shallow

shallfidd <-
read.csv("Deseq_differential_abundance_shallow_ASV_level_zero_inflated_count_model_filtered_lo
w_abundance.csv", stringsAsFactors = FALSE)

shallfilt <- shallfidd %>% filter(padj <0.05) %>%
             dplyr::select(-shallow2013,-shallow2017)

shallrest <- shallfidd %>%anti_join(shallfilt, by="sequence") %>% mutate(Phylum =
"xx_not_significant")%>%
             dplyr::select(-shallow2013,-shallow2017)

abundshall <- rbind(shallfilt,shallrest)

asvshall <- asvsSED %>% filter(site%in% "shallow")

shallfull <- abundshall %>%
            left_join(asvshall,
by=c("sequence","Kingdom","Genus","Order","Class","Family","Species"))%>%
             filter(site%in%"shallow")


###combined all

abund.all <- rbind(shallfull, interfull, deepfull)


###rest not significant
shall.not.sig <- shallrest%>%
            left_join(asvshall,
by=c("sequence","Kingdom","Genus","Order","Class","Family","Species"))%>%
             filter(site%in%"shallow")
inter.not.sig <- interrest%>%
            left_join(asvinter,
by=c("sequence","Kingdom","Genus","Order","Class","Family","Species"))%>%
             filter(site%in%"intermediate")
deep.not.sig <- deeprest%>%
            left_join(asvdeep,
by=c("sequence","Kingdom","Genus","Order","Class","Family","Species"))%>%
             filter(site%in%"deep")


abund.all.rest <- rbind(shall.not.sig, inter.not.sig, deep.not.sig)

###getting 0.5 % and others
```

```
abund.all0.5 <- abund.all%>% filter(relab >0.005)
abund.allrest <- abund.all %>%anti_join(abund.all0.5, by="sequence") %>% mutate(Phylum.x =
"xxx_Others")

###combined with others under 0.5 percent and significant over 0.5 percent

abund.all.combined <- rbind(abund.all0.5, abund.allrest)


###Want to get all non significant in one group and then others significant <0.5 % together

###signifcant within others group
sig.others <- abund.allrest %>% anti_join(abund.all.rest, by="sequence")
##not significant ones within others group
non.sig.others <- abund.all.rest %>% anti_join(sig.others, by="sequence")%>%mutate(Phylum.x
="xx_not_significant")

###combine both tables above with the tables over 0.5%

all.combined.final <- rbind(abund.all0.5,sig.others,non.sig.others)##final table


##using Genus X

abund.all$site<-factor(abund.all$site, levels = c("shallow","intermediate","deep"))

all.combined.final  %>%
ggplot(aes(x = sample, y = relab, fill =Phylum.x)) +
geom_bar( position="fill", stat="identity") +
coord_flip() +

scale_fill_manual(values=c("#db004f","#821b00","#ffa07a","#f07e00","#402300","#ffde2f","#837c00
","#adc800","#c4ff75","#01ab41","#83ffac","#dffffd","#00575d","#02affa",
"#00192b","#017fee","#00337c","#b342f4","#fbbcff","#ffa6c5","#8b004b","#00718e","#907000"))+
theme(
   legend.position = 'bottom'
  )+
  xlab('bay') + ylab('relative abundance >0.5%') +
  theme(
   axis.text.x = element_text(angle = 60, hjust = 1)
  )+
  facet_wrap(~year)+
  theme(legend.position="bottom")


only.sig <- all.combined.final %>% filter(!Phylum.x %in% c("xx_not_significant","xxx_Others"))

only.sig  %>%
ggplot(aes(x = sample, y = relab, fill =Phylum.x)) +
geom_bar( position="fill", stat="identity") +
coord_flip() +
```

```
scale_fill_manual(values=c("#db004f","#821b00","#ffa07a","#f07e00","#402300","#ffde2f","#837c00
","#adc800","#c4ff75","#01ab41","#83ffac","#dffffd","#00575d",
"#02affa","#00192b","#017fee","#00337c","#b342f4","#fbbcff","#ffa6c5","#8b004b","#00718e","#90
7000"))+
theme(
   legend.position = 'bottom'
  )+
  xlab('bay') + ylab('relative abundance >0.5%') +
  theme(
    axis.text.x = element_text(angle = 60, hjust = 1)
  )+
  facet_wrap(~year)+
  theme(legend.position="bottom")
```

#**Most changes on Genus Level, Figure 4**

```
###shallow

shallow.spec <- asvsSED %>% filter(site%in%"shallow")


UBA <- shallow.spec %>% filter(Genus%in%"UBA6092") %>%
        group_by(year, site, sample)%>%
        summarise(relab=sum(relab))%>%
        summarise(relab =mean(relab))

sul <- shallow.spec %>% filter(Genus%in%"Sulfurovum") %>%
        group_by(year, site, sample)%>%
        summarise(relab=sum(relab))%>%
        summarise(relab =mean(relab))
Gal <- shallow.spec %>% filter(Genus%in%"Gallionella") %>%
        group_by(year, site, sample)%>%
        summarise(relab=sum(relab))%>%
        summarise(relab =mean(relab))
Des <- shallow.spec %>% filter(Genus%in%"Desulfobulbus") %>%
        group_by(year, site, sample)%>%
        summarise(relab=sum(relab))%>%
        summarise(relab =mean(relab))
thau <- shallow.spec %>% filter(Genus%in%"Thauera") %>%
        group_by(year, site, sample)%>%
        summarise(relab=sum(relab))%>%
        summarise(relab =mean(relab))


first<- UBA %>%
        ggplot(aes(x=factor(site), y=relab, fill=factor(year)))+
    geom_bar(stat="identity",position=position_dodge(width=0.9), color="black")+
      scale_fill_manual(values=c("beige","antiquewhite4"))+
                ggtitle("Genus UBA6092")+
                xlab("site")+
                 ylab("relab")+
   theme(panel.grid.major = element_blank(), panel.grid.minor = element_blank(),
```

```
panel.background = element_blank(), axis.line = element_line(colour = "black"))+
    ylim(0, 0.05)

second<- CG %>%
        ggplot(aes(x=factor(site), y=relab, fill=factor(year)))+
    geom_bar(stat="identity",position=position_dodge(width=0.9), color="black")+
      scale_fill_manual(values=c("beige","antiquewhite4"))+
                ggtitle("Genus CG2-30-66-27")+
                xlab("site")+
                 ylab("relab")+
   theme(panel.grid.major = element_blank(), panel.grid.minor = element_blank(),
panel.background = element_blank(), axis.line = element_line(colour = "black"))+
    ylim(0, 0.05)


third<- sul %>%
        ggplot(aes(x=factor(site), y=relab, fill=factor(year)))+
    geom_bar(stat="identity",position=position_dodge(width=0.9), color="black")+
      scale_fill_manual(values=c("beige","antiquewhite4"))+
                ggtitle("Genus Sulfurovum")+
                xlab("site")+
                 ylab("relab")+
   theme(panel.grid.major = element_blank(), panel.grid.minor = element_blank(),
panel.background = element_blank(), axis.line = element_line(colour = "black"))+
    ylim(0, 0.05)

four<- Gal %>%
        ggplot(aes(x=factor(site), y=relab, fill=factor(year)))+
    geom_bar(stat="identity",position=position_dodge(width=0.9), color="black")+
      scale_fill_manual(values=c("beige","antiquewhite4"))+
                ggtitle("Genus Gallionella")+
                xlab("site")+
                 ylab("relab")+
   theme(panel.grid.major = element_blank(), panel.grid.minor = element_blank(),
panel.background = element_blank(), axis.line = element_line(colour = "black"))+
    ylim(0, 0.05)


five<- Des %>%
        ggplot(aes(x=factor(site), y=relab, fill=factor(year)))+
    geom_bar(stat="identity",position=position_dodge(width=0.9), color="black")+
      scale_fill_manual(values=c("beige","antiquewhite4"))+
                ggtitle("Genus Thauera")+
                xlab("site")+
                 ylab("relab")+
   theme(panel.grid.major = element_blank(), panel.grid.minor = element_blank(),
panel.background = element_blank(), axis.line = element_line(colour = "black"))+
    ylim(0, 0.05)

shal.spec <- ggarrange(first, second, third, four,five, ncol=5)


######Intermediate
```

```
inter.spec <- asvsSED %>% filter(site%in%"intermediate")


CG<- inter.spec %>% filter(Genus%in%"CG2-30-66-27") %>%
        group_by(year, site, sample)%>%
        summarise(relab=sum(relab))%>%
        summarise(relab =mean(relab))

algo <- inter.spec %>% filter(Genus%in%"Algoriphagus") %>%
        group_by(year, site, sample)%>%
        summarise(relab=sum(relab))%>%
        summarise(relab =mean(relab))
UB <- inter.spec %>% filter(Genus%in%"UBA1847") %>%
        group_by(year, site, sample)%>%
        summarise(relab=sum(relab))%>%
        summarise(relab =mean(relab))
Arc <- inter.spec %>% filter(Genus%in%"Arcobacter") %>%
        group_by(year, site, sample)%>%
        summarise(relab=sum(relab))%>%
        summarise(relab =mean(relab))
fati <- inter.spec %>% filter(Genus%in%"Desulfatiglans") %>%
        group_by(year, site, sample)%>%
        summarise(relab=sum(relab))%>%
        summarise(relab =mean(relab))


first<- CG %>%
        ggplot(aes(x=factor(site), y=relab, fill=factor(year)))+
    geom_bar(stat="identity",position=position_dodge(width=0.9), color="black")+
      scale_fill_manual(values=c("beige","antiquewhite4"))+
                ggtitle("Genus CG2-30-66-27")+
                xlab("site")+
                 ylab("relab")+
   theme(panel.grid.major = element_blank(), panel.grid.minor = element_blank(),
panel.background = element_blank(), axis.line = element_line(colour = "black"))+
    ylim(0, 0.12)

second<- algo %>%
        ggplot(aes(x=factor(site), y=relab, fill=factor(year)))+
    geom_bar(stat="identity",position=position_dodge(width=0.9), color="black")+
      scale_fill_manual(values=c("beige","antiquewhite4"))+
                ggtitle("Genus Algoriphagus")+
                xlab("site")+
                 ylab("relab")+
   theme(panel.grid.major = element_blank(), panel.grid.minor = element_blank(),
panel.background = element_blank(), axis.line = element_line(colour = "black"))+
    ylim(0, 0.12)


third<- UB %>%
        ggplot(aes(x=factor(site), y=relab, fill=factor(year)))+
    geom_bar(stat="identity",position=position_dodge(width=0.9), color="black")+
      scale_fill_manual(values=c("beige","antiquewhite4"))+
```

```
              ggtitle("Genus UBA1847")+
              xlab("site")+
               ylab("relab")+
  theme(panel.grid.major = element_blank(), panel.grid.minor = element_blank(),
panel.background = element_blank(), axis.line = element_line(colour = "black"))+
  ylim(0, 0.12)

four<- Arc %>%
      ggplot(aes(x=factor(site), y=relab, fill=factor(year)))+
   geom_bar(stat="identity",position=position_dodge(width=0.9), color="black")+
     scale_fill_manual(values=c("beige","antiquewhite4"))+
              ggtitle("Genus Arcobacter")+
              xlab("site")+
               ylab("relab")+
  theme(panel.grid.major = element_blank(), panel.grid.minor = element_blank(),
panel.background = element_blank(), axis.line = element_line(colour = "black"))+
  ylim(0, 0.12)


five<- fati %>%
      ggplot(aes(x=factor(site), y=relab, fill=factor(year)))+
   geom_bar(stat="identity",position=position_dodge(width=0.9), color="black")+
     scale_fill_manual(values=c("beige","antiquewhite4"))+
              ggtitle("Genus Desulfatiglans")+
              xlab("site")+
               ylab("relab")+
  theme(panel.grid.major = element_blank(), panel.grid.minor = element_blank(),
panel.background = element_blank(), axis.line = element_line(colour = "black"))+
   ylim(0, 0.12)

intermediate.spec <- ggarrange(first, third,second, four,five, ncol=5)

##########deep

deep.spec <- asvsSED %>% filter(site%in%"deep")


Nod<- deep.spec %>% filter(Genus%in%"Nodularia") %>%
      group_by(year, site, sample)%>%
      summarise(relab=sum(relab))%>%
      summarise(relab =mean(relab))

Desulfo <- deep.spec %>% filter(Genus%in%"Desulfobacula") %>%
      group_by(year, site, sample)%>%
      summarise(relab=sum(relab))%>%
      summarise(relab =mean(relab))
fatigl <- deep.spec %>% filter(Genus%in%"Desulfatiglans") %>%
      group_by(year, site, sample)%>%
      summarise(relab=sum(relab))%>%
      summarise(relab =mean(relab))
Drac <- deep.spec %>% filter(Genus%in%"Draconibacterium") %>%
      group_by(year, site, sample)%>%
      summarise(relab=sum(relab))%>%
```

```
        summarise(relab =mean(relab))
Algori <- deep.spec %>% filter(Genus%in%"Algoriphagus") %>%
        group_by(year, site, sample)%>%
        summarise(relab=sum(relab))%>%
        summarise(relab =mean(relab))


first<- Nod %>%
        ggplot(aes(x=factor(site), y=relab, fill=factor(year)))+
    geom_bar(stat="identity",position=position_dodge(width=0.9), color="black")+
      scale_fill_manual(values=c("antiquewhite4"))+
                ggtitle("Genus Nodularia")+
                xlab("site")+
                 ylab("relab")+
   theme(panel.grid.major = element_blank(), panel.grid.minor = element_blank(),
panel.background = element_blank(), axis.line = element_line(colour = "black"))+
    ylim(0, 0.15)

second<- Desulfo %>%
        ggplot(aes(x=factor(site), y=relab, fill=factor(year)))+
    geom_bar(stat="identity",position=position_dodge(width=0.9), color="black")+
      scale_fill_manual(values=c("beige","antiquewhite4"))+
                ggtitle("Genus Desulfobacula")+
                xlab("site")+
                 ylab("relab")+
   theme(panel.grid.major = element_blank(), panel.grid.minor = element_blank(),
panel.background = element_blank(), axis.line = element_line(colour = "black"))+
    ylim(0, 0.15)


third<- fatigl %>%
        ggplot(aes(x=factor(site), y=relab, fill=factor(year)))+
    geom_bar(stat="identity",position=position_dodge(width=0.9), color="black")+
      scale_fill_manual(values=c("beige","antiquewhite4"))+
                ggtitle("Genus Desulfatiglans")+
                xlab("site")+
                 ylab("relab")+
   theme(panel.grid.major = element_blank(), panel.grid.minor = element_blank(),
panel.background = element_blank(), axis.line = element_line(colour = "black"))+
   ylim(0, 0.15)

four<- Drac %>%
        ggplot(aes(x=factor(site), y=relab, fill=factor(year)))+
    geom_bar(stat="identity",position=position_dodge(width=0.9), color="black")+
      scale_fill_manual(values=c("beige","antiquewhite4"))+
                ggtitle("Genus Draconibacterium")+
                xlab("site")+
                 ylab("relab")+
   theme(panel.grid.major = element_blank(), panel.grid.minor = element_blank(),
panel.background = element_blank(), axis.line = element_line(colour = "black"))+
   ylim(0, 0.15)
```

```
five<- Algori %>%
       ggplot(aes(x=factor(site), y=relab, fill=factor(year)))+
    geom_bar(stat="identity",position=position_dodge(width=0.9), color="black")+
      scale_fill_manual(values=c("beige","antiquewhite4"))+
              ggtitle("Genus Algoriphagus")+
              xlab("site")+
               ylab("relab")+
   theme(panel.grid.major = element_blank(), panel.grid.minor = element_blank(),
panel.background = element_blank(), axis.line = element_line(colour = "black"))+
    ylim(0, 0.15)

deep.spec.sum <- ggarrange(first ,second,third, four,five, ncol=5)


ggarrange(shal.spec,intermediate.spec,deep.spec.sum, nrow=3)
```

#Helcom data

```
helcom <- read.csv("../../Own_Paper/Helcom/helcom.csv", stringsAsFactors = FALSE)

helcom_filt <- helcom %>%
          filter(yyyy.mm.ddThh.mm %between% c("2000-11-01T00.00","2020-11-30T00.00") )%>%
          filter(Bot..Depth..m. < 30)%>%
          filter(str_detect(yyyy.mm.ddThh.mm, "-11-"))%>%
          filter(Longitude..degrees_east. >15)%>%
          filter(Latitude..degrees_north. >55)%>%
          filter(Latitude..degrees_north. <58)

before <- helcom_filt
out <- strsplit(as.character(helcom_filt$yyyy.mm.ddThh.mm),'-')
after <- with(before, data.frame(yyyy.mm.ddThh.mm = yyyy.mm.ddThh.mm))
after <- cbind(after, data.frame(t(sapply(out, `[`))))
names(after)[2:3] <- paste("type", 1:2, sep = "_")

helcom_filt2 <- helcom_filt %>% left_join(after, by="yyyy.mm.ddThh.mm")




##Figure for different datasets

gg <- ggplot(helcom_filt2, aes(x=as.numeric(type_1), y=as.numeric(helcom_filt2$DOXY..ml.l.),
color=as.numeric(helcom_filt2$Bot..Depth..m.)))+
   geom_point(stat="identity",position="identity")+
     theme(axis.text.x = element_text(angle = -90, hjust = 0, vjust =0.5))+
  scale_color_gradient(low="yellow",high = "darkblue")+
  geom_smooth(method="lm")+
 ylim(0,13)+
  xlim(2000, 2019)+
  stat_cor(label.y=11) +
  stat_regline_equation(label.y=10)
 gg
```

#Oxygen 2017

```
meta.all <- read.csv("meta_SED_TS_Oestanvik.csv")

meta.all$month <- factor(meta.all$month, levels =
c("march","may","june","july","august","october","november","december"))

oxy <-  meta.all %>%
  filter(year==c("2017"))%>%
  ggplot(aes(x=month, y=oxygen1, color=site, shape=site, group=site))+
               geom_point(size=4)+
               geom_line()+
               scale_color_manual(values=c("black","darkolivegreen4","darksalmon"))+
               ggtitle("Oxygen")+
               xlab("date")+
                ylab("mg/L") +
                 theme(
                     axis.text.x = element_text(angle = 25, hjust = 1))+
              geom_hline(yintercept=2, linetype="dashed", color = "grey", size=1)+
   theme(panel.grid.major = element_blank(), panel.grid.minor = element_blank(),
panel.background = element_blank(), axis.line = element_line(colour = "black"))
```

#SIMPER analysis

```
#based on GENUS level counts
#Filter the samples (groups) you are interested in to compare in SIMPER, as well as summarise the counts on Genus level
GenusSED <- asvsSED %>%
  filter(month=="november")%>%
  filter(year %in%c("2017"))%>%
  filter(!sample %in% c("E6","E8","E3"))%>%
  filter(!site %in% c("shallow"))%>%
  group_by(Genus,sample)%>%
  summarise(count = sum(count))%>%
  ungroup()%>%
  filter(!Genus %in% "Unclassified")%>%
  spread(Genus,count, fill= 0) %>%
  remove_rownames() %>%
  column_to_rownames(var = "sample")

#Metatable used to distinguish between the groups, you want to compare
metasubSED_sub <- metasubSED %>%
  filter(!site %in% "shallow")%>%
  filter(year %in% "2017")

#Simper analysis
(sim <- with(metasubSED_sub , simper(GenusSED, site)))

#Summary of the analysis
"summary"(sim, ordered=TRUE)

#Make a data.frame of the simper output
sim.data <- data.frame(unclass(summary(sim)), check.names = FALSE, stringsAsFactors = FALSE)


##Overall dissimilarity between bacterial communitites based on SIMPER analysis
lapply(sim, FUN=function(x) {x$overall})
```
